# Fine‐scale barriers to connectivity across a fragmented South‐East Asian landscape in six songbird species

**DOI:** 10.1111/eva.12918

**Published:** 2020-03-05

**Authors:** Emilie Cros, Elize Y. X. Ng, Rachel R. Y. Oh, Qian Tang, Suzan Benedick, David P. Edwards, Suzanne Tomassi, Martin Irestedt, Per G. P. Ericson, Frank E. Rheindt

**Affiliations:** ^1^ Department of Biological Sciences National University of Singapore Singapore Singapore; ^2^ Centre for Urban Greenery and Ecology National Parks Board Singapore Singapore; ^3^ School of Biological Sciences University of Queensland Brisbane QLD Australia; ^4^ Sustainable Agriculture School Universiti Malaysia Sabah Sabah Malaysia; ^5^ Department of Animal and Plant Sciences University of Sheffield Sheffield UK; ^6^ Department of Bioinformatics and Genetics Swedish Museum of Natural History Stockholm Sweden; ^7^ Department of Zoology Swedish Museum of Natural History Stockholm Sweden

**Keywords:** barriers, birds, connectivity, conservation genetics, fragmentation, RADseq, tropics

## Abstract

Habitat fragmentation is a major extinction driver. Despite dramatically increasing fragmentation across the globe, its specific impacts on population connectivity across species with differing life histories remain difficult to characterize, let alone quantify. Here, we investigate patterns of population connectivity in six songbird species from Singapore, a highly fragmented tropical rainforest island. Using massive panels of genome‐wide single nucleotide polymorphisms across dozens of samples per species, we examined population genetic diversity, inbreeding, gene flow and connectivity among species along a spectrum of ecological specificities. We found a higher resilience to habitat fragmentation in edge‐tolerant and forest‐canopy species as compared to forest‐dependent understorey insectivores. The latter exhibited levels of genetic diversity up to three times lower in Singapore than in populations from contiguous forest elsewhere. Using dense genomic and geographic sampling, we identified individual barriers such as reservoirs that effectively minimize gene flow in sensitive understorey birds, revealing that terrestrial forest species may exhibit levels of sensitivity to fragmentation far greater than previously expected. This study provides a blueprint for conservation genomics at small scales with a view to identifying preferred locations for habitat corridors, flagging candidate populations for restocking with translocated individuals and improving the design of future reserves.

## 1 INTRODUCTION

Habitat fragmentation is amongst the main drivers of species extinction (Hughes, 2017). The global effects of habitat fragmentation are slated to intensify in the future with current trends in human population growth and development (Sodhi, Liow, & Bazzaz, 2004). Yet the adverse effects of fragmentation remain difficult to characterize, let alone quantify, as they differ in severity depending on species and spatial arrangements (Bregman, Sekercioglu, & Tobias, 2014; Keinath et al., 2017; Visco et al., 2015), making it crucial to examine the impacts of fragmentation on species spanning a range of life histories.

In fragmented landscapes, subpopulations may become completely isolated from one another depending on the distance among patches, the suitability of the intervening habitat matrix and the species’ dispersal capability. A lack of gene flow and connectivity among subpopulations increases the probability of inbreeding and stochastically induced loss of genetic diversity, with a potentially detrimental impact on population fitness, as inbreeding may lead to the expression of deleterious genetic effects and low genetic diversity may hamper adaptation to environmental change (Frankham, Ballou, & Briscoe, 2010). To prevent local extinction, information on connectivity and gene flow is therefore essential.

A major constraint in gauging impacts of fragmentation on specific organisms has been a lack of empirical data on a species’ actual dispersal capabilities, particularly in fragmented tropical environments (Sodhi, Şekercioğlu, Barlow, & Robinson, 2011; Visco et al., 2015). Most experimental studies have used patch occupancy patterns or translocation experiments, which are not representative of natural dispersal conditions (Keinath et al., 2017; Laurance & Gomez, 2005; Sodhi et al., 2011; Visco et al., 2015). Although some individuals may be able to occasionally cross barriers, the level of gene flow among populations might still be limited (Proctor, McLellan, Strobeck, & Barclay, 2005). Molecular techniques such as next‐generation sequencing, which has recently been used to reveal fine‐scale population structure (Kjeldsen et al., 2016; Szulkin, Gagnaire, Bierne, & Charmantier, 2016), offer new promise in investigating the influence of fragmentation and barriers on population connectivity (Angeloni, Wagemaker, Vergeer, & Ouborg, 2012; Sawaya, Kalinowski, & Clevenger, 2014) at ever‐finer geographic scales.

Because of their flight ability, birds are often thought to be comparatively little impacted by fragmentation. Yet habitat gaps are known to affect movements of understorey specialist birds among forest patches (Laurance & Gomez, 2005) and understorey insectivores have been shown to be among the first biotic groups to be extirpated from forest fragments after fragmentation (Laurance et al., 2011). Insectivorous birds’ vulnerability to habitat fragmentation has sometimes been ascribed to their territoriality and poor dispersal capability (Castelletta, Sodhi, & Subaraj, 2000; Visco et al., 2015). One would therefore expect more dispersive species to be less affected by fragmentation, although even species with high dispersal capabilities are reported to be adversely affected by fragmentation (Ferraz et al., 2007).

In this study, we examined patterns of gene flow and connectivity across subpopulations of six passerine birds in a heavily fragmented forest landscape on the densely urbanized island of Singapore. Our panel of species is characterized by differences in levels of forest dependence and spans a variety of diets and forest strata. Using thousands of genome‐wide single nucleotide polymorphisms (SNPs) in combination with multiple population‐genomic approaches, we investigated how life history and ecological requirements affect a species’ response to fragmentation on a heavily urbanized tropical rainforest island.

## 2 MATERIALS AND METHODS

### 2.1 Samples and study sites

**Figure 1:**
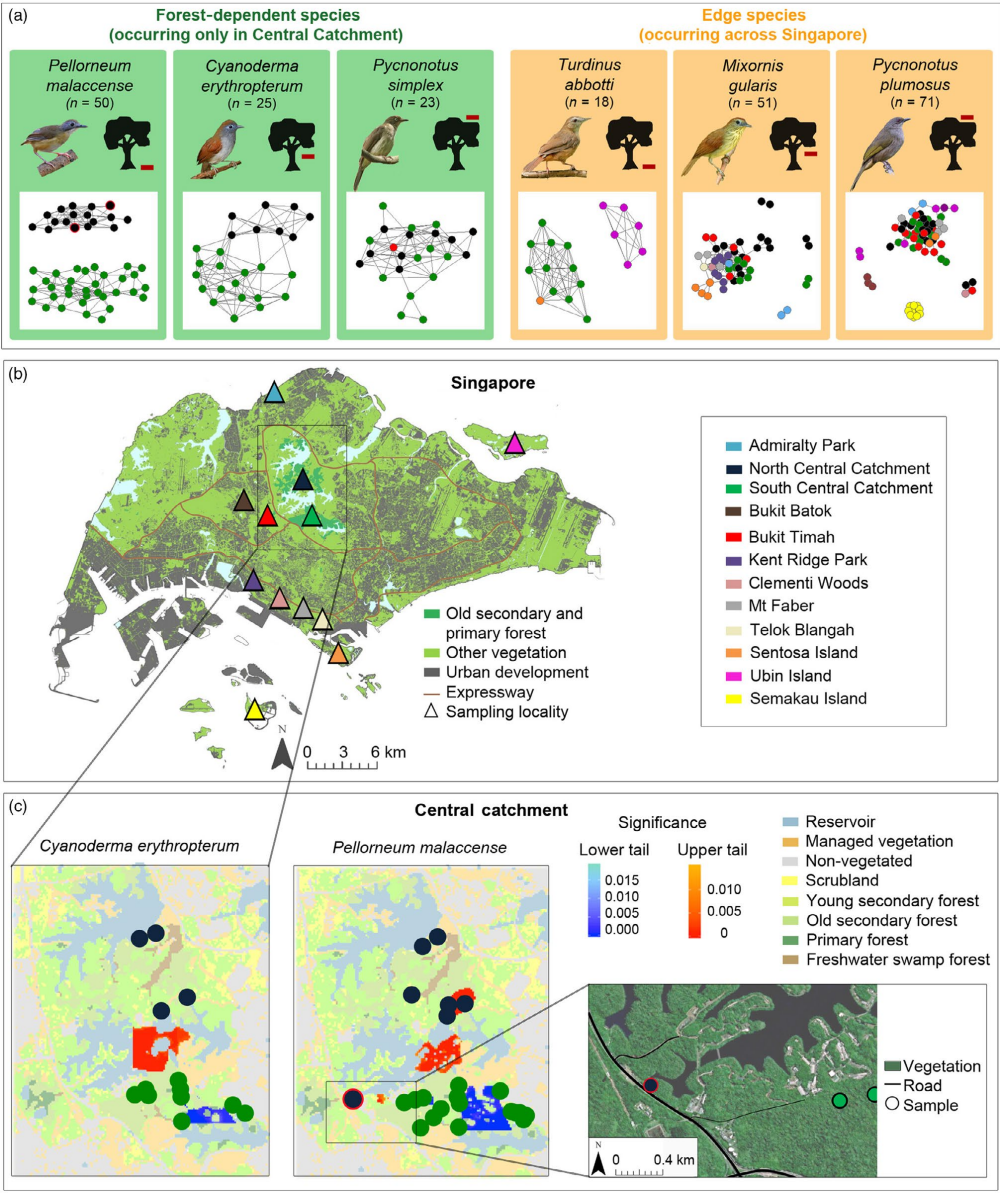
Population structure of *Pellorneum malaccense*, *Cyanoderma erythropterum*, *Turdinus abbotti*, *Mixornis gularis*, *Pycnonotus plumosus* and *Pycnonotus simplex* across Singapore. (a) Population clustering based on network analysis at k = 10. Each dot represents one sample coloured according to sampling locality (see panel b). Red horizontal bars next to the black trees indicate forest stratum. Bird pictures courtesy of Daniel Koh; (b) Singapore map and sampling localities (adapted from Yee et al., 2011). (c) Barriers to and conduits for gene flow in the forest‐dependent *C. erythropterum* and *P. malaccense* in the Central Catchment using spatially explicit individual‐based analysis. Areas dominated by pairwise genetic divergences significantly higher (*p* < .05) than assumptions of isolation by distance are shown in hues of red and indicate the presence of barriers. A genetic similarity significantly higher than expected from isolation by distance is shown in hues of blue, indicating areas between relatively closely related individuals. Dot colour (green vs. black) corresponds to sampling localities following panel b

Using mist‐netting across the South‐East Asian island nation of Singapore, including its smaller offshore islands of Sentosa and Ubin (Figure 1b), we collected blood samples of four insectivorous babblers (families Timaliidae and Pellorneidae sensu Moyle, Andersen, Oliveros, Steinheimer, and Reddy (2012)): two forest‐dependent understorey species (*Pellorneum malaccense* (Short‐tailed Babbler) and *Cyanoderma erythropterum* (Chestnut‐winged Babbler)) and two edge‐tolerant species (*Turdinus abbotti* (Abbott's Babbler) and *Mixornis gularis* (Striped Tit‐babbler)) (Table S1). Moreover, we collected blood samples of two frugivorous bulbuls (family Pycnonotidae), one forest‐dependent species (*Pycnonotus simplex* (Cream‐vented Bulbul)) and one edge‐tolerant species (*Pycnonotus plumosus* (Olive‐winged Bulbul)). Mist‐netting and sampling in Singapore were conducted in accordance with Institutional Animal Care and Use Committee regulations (B13‐4738/ B16‐0572) (permit NP/RP13‐019‐2). We also collected blood of *P. malaccense* and *C. erythropterum* from Danum Valley (Sabah, Malaysian Borneo) for comparisons of genetic diversity (Table S2) adhering to the stipulations of the Sabah Biodiversity Enactment (2000) (permits JKM/MBS.1000‐2/2JLD.3(118), JHL100.7/27 and JKM/MBS.1000‐2/3JLD.2(65)). Additional blood/tissue samples from the main island of Singapore, including from the nearby islet of Semakau, from northern Borneo (Sarawak, Malaysia) and Vietnam were provided by museums (see Acknowledgements; Tables S1 and S2). We also included sequence data of 37 *M. gularis* which were obtained from a previous study (Tan et al., 2018).

### 2.2 Extraction and ddRADseq library preparation

DNA extractions were performed using the DNeasy Blood & Tissue Kit (QIAGEN, Hilden, Germany) following the manufacturer's protocols for blood or tissue. We prepared five libraries following Ng et al.’s (2017) double‐digest restriction‐associated DNA sequencing (ddRADseq) protocol. During library preparation, the last clean‐up step was replaced by a size selection step to ensure size requirements would be met. To select for 250–600 bp fragments, as well as for the clean‐up steps, we used Sera‐Mag magnetic beads (Thermo Scientific). DNA quantifications were performed with a Qubit 2.0 Broad Range DNA Assay (Invitrogen). Before pooling samples, we checked DNA fragment size using a Fragment Analyser (Advanced Analytical Technologies, Inc.). The five libraries were then spiked with 5% PhiX to prevent low nucleotide diversity issues to affect the quality of the data and subsequently sequenced on an Illumina HiSeq 2500 platform (150 bp paired‐end run) at the Singapore Centre for Environmental Life Sciences and Engineering. Some of the *P. plumosus* samples were extracted with an Exgene Clinic SV kit (GeneAll Biotechnology) and were sequenced in one additional library by a different provider (BGI Tech Solutions Co., Ltd) on a 100 bp paired‐end run on an Illumina HiSeq 2000 platform. This library was prepared following Tan et al.’s (2018) protocol. Restriction and ligation were conducted in a single step with duplicates for each sample, and PCRs were carried out with 18 cycles and triplicates for each sample (Tan et al., 2018).

### 2.3 SNP calling

Reads were demultiplexed and trimmed to 145 bp (95 bp for *M. gularis* and *P. plumosus*) with “process_radtags” in STACKS 1.42 (Catchen, Hohenlohe, Bassham, Amores, & Cresko, 2013). Reads with one or more uncalled bases were removed. Files were renamed using bbmap v35.85 (Bushnell, 2016), whereupon reads were aligned to the *M. gularis* whole genome (Tan et al., 2018) using bwa 0.7.13 (Li, 2013). We used samtools 1.3.1 to convert sam files to bam files and to filter files with a minimum required MAPQ score of 20 (Li et al., 2009). To call and filter SNPs we used “ref_map.pl” and “population” in STACKS 1.42. We set stack depth to 10 and the percentage of individuals represented at each locus to 0.9 and admitted only one random SNP per locus to preclude analysis of linked SNPs. We checked for SNPs under selection using BayeScan2.1 (Foll & Gaggiotti, 2008) and used Plink 1.9 to test for linkage disequilibrium among loci and to calculate the level of missing data (Chang et al., 2015). Two sets of unlinked SNPs were generated for each species: one with all samples and one in which we removed close kin. For the latter SNP sets, we randomly removed one individual per pair for which comparisons revealed a kinship coefficient higher than 0.10 (see section 2.4). This latter SNP set was used for STRUCTURE analysis (Pritchard, Stephens, & Donnelly, 2000) and to control for the impact of relatedness on analyses such as PCA or on summary statistics such as *F*_ST_.

To compare genomic diversity of the fragmented population from Singapore to populations from contiguous blocks of unfragmented forests elsewhere, we created an additional set of unlinked SNPs for each species (except *T. abbotti*). We randomly subsampled individuals from Singapore to ensure equal representation of samples for compared localities and further controlled for sampling area size between compared localities.

### 2.4 Genomic diversity

Average expected and observed heterozygosity for each population were calculated using the R package adegenet 1.3‐1 (Jombart, 2008; Jombart & Ahmed, 2011). We estimated relatedness using Identity‐By‐Descent with the R package SNPRelate 1.6.6 (Zheng et al., 2012). Inbreeding coefficients were calculated using COANCESTRY (Wang, 2011). To select the most suitable estimator for each species, simulations were conducted with the estimators implemented in COANCESTRY (Brekke, Bennett, Wang, Pettorelli, & Ewen, 2010), specifying 4,200 individuals with inbreeding coefficients ranging from 0 to 1, at 0.05 intervals, with 100 individuals for each inbreeding category. Allele frequencies and missing data used for these simulations were calculated based on SNP loci generated from Plink. The best estimator for inbreeding across species was either the dyadic (DyadML) or triadic likelihood estimator (TrioML).

### 2.5 Population structure analysis

To identify potential population subdivision within Singapore, we used the Bayesian clustering method in STRUCTURE v. 2.3.4 (Pritchard et al., 2000). Ten replicates of each *K* (1–10) were run using 50,000 burn‐in steps and 100,000 Markov chain Monte Carlo samples. We identified the number of genetic clusters which best partitions the data using Evanno, Regnaut, and Goudet’s (2005) delta *K* method. As the inferred *K* value is not always biologically meaningful, we always investigated results obtained across different *K* values (Pritchard et al., 2000). We also performed principal component analysis (PCA) using the R package SNPRelate 1.6.6 (Zheng et al., 2012). Additionally, to investigate fine‐scale population structure, we used the network clustering method NetView (Neuditschko, Khatkar, & Raadsma, 2012; Steinig, Neuditschko, Khatkar, Raadsma, & Zenger, 2016) as implemented in R, assessing population structure at different maximum numbers of nearest neighbours (*k*) an individual can have. Individuals that have the smallest genetic distance are connected according to a threshold that varies with *k*. The level of genetic differentiation between identified clusters for each species was assessed by computing Nei's pairwise *F*_ST_ and its significance with the R package adegenet 1.3‐1 (Jombart, 2008; Jombart & Ahmed, 2011).

### 2.6 Spatial analysis

We used a spatially explicit, individual‐based approach (DResD) as implemented in R to investigate potential barriers to or conduits for gene flow (Keis et al., 2013). Based on a geographic and genetic distance matrix, this approach calculates the geographic distribution of isolation‐by‐distance (IBD) residuals to identify areas for which genetic differences among individuals appear significantly lower or higher than the IBD trend would predict, indicating potential barriers or corridors to gene flow, respectively. For this procedure, mid‐points between each sample pair were used to estimate the inverse distance‐weighted average IBD residuals over a rectangular grid covering the geographic area of sampling. The statistical significance and power were tested using 1,000 randomization iterations and 250 bootstrap iterations. We discarded cells with power < 0.8 as not statistically significant to control for type II errors (Aberson, 2010). We then investigated the level of resistance of landscape features for each species. For the resistance map, we extracted the weighted average IBD residuals from the DResD analysis for each cell. For the landscape map, we used a classification of landscape features according to Yee, Corlett, Liew, and Tan (2011). We rasterized the resistance and landscape maps into a 696 × 803 grid (each grid cell 50m × 50m). Using both maps we then identified the level of resistance of each landscape feature.

Using the same geographic and genetic matrix, we computed spatial autocorrelation between individuals using 999 permutations and bootstraps in GenAlEx 6.5 (Peakall & Smouse, 2006, 2012). We investigated the correlation r across different distance classes to compute the genetic patch size for each species (Peakall, Ruibal, & Lindenmayer, 2003; Peakall & Smouse, 2012). Euclidean genetic distances were obtained using the R package adegenet 1.3‐1 (Jombart, 2008; Jombart & Ahmed, 2011). Only individuals with precise GPS information were used.

## 3 RESULTS

### 3.1 Summary statistics

We successfully obtained sequence data for 218 individuals across Singapore with an average number of 3.7 million reads per individual and an average of ~ 17,245 SNPs per species complex (see Table 1 for summary statistics). Overall 10 samples were discarded during quality control: three *P. malaccense* for which sequencing failed; and seven *P. plumosus* with 18%–80% missing data. None of the loci exhibited a signature of selection. Average expected heterozygosity was ~ 20%–100% higher in babbler than in bulbul species (Table 1). We identified closely related kin in all species; the number of pairwise kinship comparisons exceeding 0.10 was between two to six times higher for babblers than bulbuls (Table 1). Inbreeding coefficients across Singapore were generally low for all species (<0.1), with the exception of *P. simplex* (up to 0.15; Table 1).

**Table 1:** Summary statistics of the six study species for both the data set with and without kin

	Number of samples	Average read number per individual	Number of SNPs	He^a^	Ho^b^	Average inbreeding coefficient	Inbreeding coefficient range	Number (percentage) of pairwise kinship comparison ≥ 0.10	Dataset with removal of kin	
									Number of samples	Number of SNPs
*Pellorneum malaccense*	50	2,947,100	16,614	0.28	0.27	0.027^c^	0–0.17	23/1,525 (1.5%)	36	16,422
*Cyanoderma erythropterum*	25	2,728,770	12,733	0.32	0.32	0.013^d^	0–0.09	8/300 (2.7%)	19	12,537
*Turdinus abbotti*	18	2,952,598	16,223	0.29	0.28	0.046^c^	0–0.15	5/153 (3.3%)	14	15,909
*Mixornis gularis*	51	6,395,537 (8,086,118 in the 100‐bp run; 2,914,931 in the 150‐bp run)	24,914	0.24	0.22	0.063^c^	0.011–0.22	13/1,275 (1%)	40	24,521
*Pycnonotus simplex*	23	3,167,172	16,911	0.19	0.17	0.134^d^	0.065–0.20	1/253 (0.4%)	22	16,796
*Pycnonotus plumosus*	71	3,802,724 (5,871,214 in the 100‐bp run; 2,866,978 in the 150‐bp run)	16,079	0.16	0.14	0.083^d^	0.004–0.20	13/2,485 (0.5%)	61	15,638

a Average expected heterozygosity.

b Average observed heterozygosity.

c Triadic likelihood estimator.

d Dyadic likelihood estimator.

### 3.2 Population genetic diversity

Genetic diversity of fragmented Singaporean populations of the edge‐tolerant *M. gularis* and *P. plumosus* as well as the forest‐dependent *P. simplex* was similar to that of populations from contiguous forests in Vietnam or Borneo (Table 2). In contrast, genetic diversity of populations of the forest‐dependent *P. malaccense* and *C. erythropterum* in contiguous Bornean forests was 1.6 to 3.2 times higher than that of Singaporean populations (Table 2).

**Table 2:** Genetic diversity for *Pellorneum malaccense*, *Cyanoderma erythropterum*, *Mixornis gularis*, *Pycnonotus plumosus* and *Pycnonotus simplex* populations from Singapore and from large contiguous forest areas outside Singapore

Species	SNP number	Singapore			Contiguous forest outside Singapore		
		Number of samples	H_o_ a	H_e_ b	Number of samples	H_o_ a	H_e_ b
*P. malaccense* f	17,174	14	0.06	0.06	14	0.17^c^	0.19^c^
*C. erythropterum* f	13,954	6	0.14	0.13	6	0.20^c^	0.21^c^
*M. gularis* g	19,301	8	0.18	0.19	8	0.18^d^	0.19^d^
*P. simplex* f	13,497	14	0.12	0.14	14	0.12^e^	0.14^e^
*P. plumosus* g	10,017	4	0.25	0.25	4	0.25^e^	0.25^e^

a Average observed heterozygosity.

b Average expected heterozygosity.

c Sabah.

d Vietnam.

e Sarawak.

f Forest‐dependent species.

g Edge‐tolerant species.

### 3.3 Population structure

We found strong evidence for the presence of two separate population clusters within Singapore's last large remaining forest reserve (Central Catchment) in the forest‐dependent *P. malaccense* and *C. erythropterum* (Figure 1a, Figures S1 and S2). While *C. erythropterum* seems to have retained a higher level of recent connectedness between its two populations than *P. malaccense*, both species were characterized by an unusually clean division between two population clusters separated by often less than 1 km (Figure 1b, c).

In contrast, the forest‐dependent *P. simplex* as well as the edge‐tolerant *T. abbotti*, *M. gularis* and *P. plumosus* displayed no spatial clustering across the main island of Singapore (Figure 1a, Figures S3–S6). Outliers were present in both NetView analysis and PCA for *M. gularis* and *P. plumosus* (Figure 1a, Figures S5 and S6) and persisted in PCA after kin removal (not shown). Subpopulations of *T. abbotti*, *M. gularis* and *P. plumosus* from Sentosa Island clustered with main island populations and only separated in *T. abbotti* under additional exploration of the less significant principal component 4 (Figure 1a, Figures S4–S6). Ubin Island subpopulations clustered separately from main island subpopulations for *T. abbotti* but not for *P. plumosus*. Only the more distant subpopulation from the islet of Semakau clustered separately for *P. plumosus*. Interestingly for *P. plumosus*, most main island samples appeared closer to the Semakau Island subpopulation than to a number of select outliers on the main island itself (Figure S6).

Pairwise F_ST_ comparisons generally corroborated the discrete population clusters shown in population‐genomic analyses (Figure 1a, Figures S1‐S6) by revealing significant genetic differentiation among geographic subpopulations (Table 3). Corresponding pairwise F_ST_ values for the data sets in which close kin had been removed were similar, albeit slightly higher (not shown).

**Table 3:** Pairwise F_ST_ and its significance for *Pellorneum malaccense*, *Cyanoderma erythropterum*, *Turdinus abbotti* and *Pycnonotus plumosus*

Species	Comparison	Pairwise *F*_ST_ (p value)
*P. malaccense*	Northern vs. southern Central Catchment	0.0275 (*p* < .001)
*C. erythropterum*	Northern vs. southern Central Catchment	0.0476 (*p* < .001)
*T. abbotti*	Main Island vs. Ubin Island	0.084 (*p* < .001)
*P. plumosus*	Main Island (incl. Ubin) vs. Semakau Island	0.020 (*p* < .001)

### 3.4 Spatial analysis

The spatially explicit DResD analysis revealed fine‐scale genetic isolation in some species (Figure 1c, Figure S7). A barrier to gene flow was detected within the Central Catchment in the forest‐dependent *C. erythropterum* and *P. malaccense* (Figure 1c). Open areas such as reservoirs, scrubland and/or man‐made structures were identified as potential barriers to gene flow in those species, whereas old secondary and primary forests seem to act as corridors (Figure S8). In both species, genetic similarity among samples within the southern part of the reserve was significantly higher than expected under isolation by distance, which may indicate an impoverished gene pool in an isolated population.

Areas of high genetic dissimilarity, corresponding to areas where genomic outliers (possible immigrants) were sampled, were observed in *M. gularis* and *P. plumosus* (Figure S7). After removal of outliers, we still detected a barrier to gene flow between subpopulations from the main island and the more distant islet of Semakau as well as a corridor between the main island and adjacent Ubin Island for *P. plumosus*, suggesting that open sea is not a barrier per se and only larger water barriers limit connectivity in this edge‐tolerant species (Figure S7). A sampling gap between the main island and Ubin Island may have prevented us from further narrowing down the landscape features conducive to gene flow in this species (Figure S8). Similarly, a barrier between northern/central and southern subpopulations as well as a corridor within southern subpopulations was detected for *M. gularis* (Figure S7). In this species, scrubland seems to be an efficient corridor, whereas open and built‐up areas appear to limit connectivity. Based on lower sample sizes, spatial data for *T. abbotti* did not pass the conservative power threshold with the exception of a small area within the Central Catchment for which resistance computations suggested that at least open water may act as a barrier (data not shown). No significant barriers were found in *P. simplex* (data not shown).

Spatial autocorrelation indicated small genetic patch sizes of 2.5 and 4 km for the forest‐dependent *C. erythropterum* and *P. malaccense*, respectively, indicating poor dispersal capability (Figure S9). Edge‐tolerant species, in contrast, showed larger genetic patch sizes: 8 km for *M. gularis*, 15 km for *T. abbotti* and 18 km for *P. plumosus* (Figure S9). The genetic patch size emerged as larger than the sampling area for *P. simplex*, so we could not obtain an estimate.

## 4 DISCUSSION

In this study, we used ~ 13,000–25,000 SNPs per songbird species to investigate population genetic diversity, inbreeding, gene flow and connectivity in four insectivorous babblers (two forest‐dependent and two edge‐tolerant) and two frugivorous bulbuls (one forest‐dependent and one edge‐tolerant) across a heavily fragmented and urbanized tropical forest landscape in Singapore. The strength of our data set is that it compares the genomic signatures of more widespread generalist species with those of habitat specialists of a formerly widespread distribution that have – by now – become restricted to the last big forest reserve of the island. While populations of the forest‐dependent *P. simplex* as well as the three edge‐tolerant species, *T. abbotti*, *M. gularis* and *P. plumosus*, are well connected across the main island of Singapore, the two forest‐dependent understorey insectivorous babblers, *P. malaccense* and *C. erythropterum*, appear to be deeply affected by fragmentation, underscoring that understorey insectivores are more strongly affected by fragmentation than other birds (Laurance et al., 2011).

### 4.1 Fragmentation leads to differential loss of population genetic diversity

The population genetic diversity of *M. gularis*, *P. plumosus* and *P. simplex* appears comparable between Singapore Island and contiguous forest blocks of a similar size in Sarawak and Vietnam (Table 2). In contrast, genetic diversity of the understorey insectivores *P. malaccense* and *C. erythropterum* was up to three times higher for populations from the contiguous forest block around Danum Valley (Sabah, Malaysia) than for the Central Catchment in Singapore – despite the comparable size of Singapore's Central Catchment and the area in Danum Valley where the samples had been obtained. This dramatic reduction of population genetic diversity in Singapore's fragmented landscape is consistent with results obtained from mitochondrial analysis of *P. malaccense* (Sadanandan & Rheindt, 2015) as well as the Banded Langur *Presbytis femoralis* (Ang, Srivasthan, Md‐Zain, Ismail, & Meier, 2012), suggesting that urbanization over only half a century (Ng, Corlett, & Tan, 2011) can drastically deplete a species’ pool of genetic diversity. An alternative explanation for this pronounced discrepancy in diversity would invoke Singapore's small‐island status as compared with Borneo's immense landmass. However, Singapore is only ~ 700 m from continental Asia and has been connected throughout ~ 91% of the past 250,000 years (Voris, 2000). Additionally, if Singapore's small‐island status played a large role, we would expect comparable declines in genetic diversity in the more generalist species of our panel, at least one of which (*M. gularis*) was shown to be a relatively poor disperser (Figures S7 and S9; also see Tan et al., 2018). Therefore, while Singapore's island status may have contributed marginally to lower genetic diversity, it cannot explain the magnitude of diversity loss in Singapore.

Our analysis provides evidence for limited connectivity between the northern and southern parts of Singapore's largest forest reserve in both forest‐dependent understorey insectivores (Figure 1). The northern and southern subpopulations are each restricted to patches of 935 and 483 ha, respectively (Castelletta et al., 2000). Reduction in population size and diversity loss are well‐established factors leading to inbreeding and increasing stochastic extinction risk (Evans & Sheldon, 2008; Huang, Hauert, & Traulsen, 2015; Spielman, Brook, & Frankham, 2004; Visco et al., 2015), including in Singapore (Tan et al., 2018). The Central Catchment in Singapore has recently lost similar avian understorey insectivores following a gradual, stepwise decline (Yong, 2009), and fragmentation through road construction has been associated with faunal local extinctions in the same area (Davison & Chew, 2019). In the context of our two babbler species, we detected high genetic similarity among individuals in the smaller, southern part of the reserve (Figure 1); and although our calculation of summary statistics for inbreeding did not yield significant levels, the large number of closely related kin identified within each fragment (Table 1) and the small size of those fragments conclusively point to an impending increase in population‐wide homozygosity, a known correlate of extinction risk (Evans & Sheldon, 2008; Spielman et al., 2004; Visco et al., 2015).

### 4.2 Mapping connectivity across species with different life histories

This study is one of the first based on genome‐wide DNA and extremely fine‐scale geographic sampling to show that species biology and life history closely determine dispersal capability. The forest‐dependent *P. malaccense, C. erythropterum* and *P. simplex* are all restricted to Singapore's largest forest patches but differ in their response to fragmentation. The two understorey insectivores, *P. malaccense* and *C. erythropterum*, emerged as particularly sensitive to habitat fragmentation. Built‐up areas and reservoirs may act as especially strong barriers to their dispersal (Figure 1, Figure S8), corroborating that open space can reduce or even prevent the movement of forest‐dependent understorey birds (Laurance & Gomez, 2005). While *P. malaccense* lives on the forest floor, *C. erythropterum* frequents the lower to mid‐stratum (Wells, 2007). This slight difference in microhabitat may explain why *P. malaccense's* genetic separation across small geographic scales appears to be even stronger than in *C. erythropterum* (Figure 1a).

Unlike the two understorey babblers, the forest‐dependent *P. simplex*, a canopy frugivore, does not seem to be noticeably affected by the presence of habitat gaps or highways, as exemplified by the absence of population subdivision across Singapore. This is consistent with previous results based on 40 Amazonian birds indicating that canopy species have a greater dispersal propensity and a lower rate of diversification than understorey species (Burney & Brumfield, 2009).

While forest specialists are restricted to larger forest patches, edge species such as *T. abbotti*, *M. gularis* and *P. plumosus*, whether insectivorous or frugivorous, occur more or less island‐wide and appear to be well connected by gene flow. *Mixornis gularis* and *P. plumosus* may be favoured at least temporarily by fragmented landscapes and the resulting increase in edge habitat (Moradi & Mohamed, 2010). Previous mitochondrial analysis of *P. plumosus* in Singapore suggested a recent population expansion (Tang, Sadanandan, & Rheindt, 2015). Only larger water bodies seem to act as a barrier capable of affecting gene flow among subpopulations of *P. plumosus* (Table 3, Figure S7). However, although the sea barrier of 6.51 km separating the islet of Semakau from Singapore seems to have led to population separation (Figure 1a, Table 3, Figure S6), the sea is not an absolute obstacle (Figures S6 and S8), as we detected population‐genomic outlier individuals of *P. plumosus* on Singapore that are possibly migrants from more distant Sundaic islands, such as the Riau Archipelago. Although edge species appear well connected across large areas, fragmentation may still compromise their connectivity and recolonization potential over a longer time scale. For example, despite a well‐connected network of subpopulations of *M. gularis* across the island (Figure 1a), we noted dispersal capabilities half as high as those of *T. abbotti* and *P. plumosus* and detected a noticeable dispersal barrier within Singapore (Figures S7 and S9).

### 4.3 Detection of fine‐scale barriers to gene flow

Within Singapore's largest forest reserve, analyses across two different avian insectivores of the undergrowth consistently identified reservoirs and adjacent areas of nonforest vegetation, such as golf courses, as barriers to gene flow (Figure 1c), underscoring the importance of creating corridors of suitable habitat to counteract fragmentation. In *P. malaccense*, in particular, our dense sampling regime allowed us to identify a forest area where both subpopulations (northern and southern Central Catchment) most closely approach each other. The area in question happens to be near the only site where forests north and south of the main dividing reservoir are connected along a narrow nexus, delimited on the west by a large highway (Figure 1c). The deep population division between the northern and southern subpopulations shows that this habitat nexus, running between the reservoir and the large highway, is insufficient for gene flow, indicating that a building development across ~ 180 m and small roads (~4 m wide) are effective in limiting connectivity between populations of sensitive understorey insectivores (Figure 1c).

This pattern contrasts with translocation studies demonstrating that understorey birds have been able to cross highways or gaps to return to their home range (Laurance & Gomez, 2005). Dispersal capabilities of the birds studied in those experiments may exceed those of *P. malaccense* and *C. erythropterum*. For instance, *P. malaccense* is rarely observed within 25m of forest edge (Zakaria, Rajpar, Moradi, & Rosli, 2014) and might not be able to use narrow remnant forest strips. Alternatively, although understorey specialists may be capable of occasional or rare dispersal events, those may not suffice to counteract fragmentation effects. Lastly, a translocated individual's motivation to return to its home range might not be representative of spontaneous, natural dispersal.

Man‐made barriers such as highways have been shown to lead to clear population differentiation in less than 20 years in a wide range of terrestrial species (voles: Gerlach & Musolf, 2000; ground beetles: Keller & Largiadèr, 2003; bears: Proctor et al., 2005; coyotes/bobcats: Riley et al., 2006). However, to our knowledge this is the first time that genomic evidence identifies anthropogenic structures as obstacles to gene flow in birds at such fine‐scale resolution. Our analysis reveals ongoing population fragmentation within a reserve of only 10km^2^ size.

### 4.4 Genomics as a tool for conservation

Genome‐wide markers allowed us to identify extremely fine‐scale geographic barriers to connectivity and to characterize dispersal capability. Using a panel of six songbird species, some landscape features such as reservoirs and man‐made structures were shown to drastically compromise connectivity in understorey insectivores, underscoring the usefulness of genomic tools in understanding the impact of fragmentation on population connectivity. Importantly, we demonstrate that these landscape barriers have adverse impacts on connectivity not only on IUCN‐listed species such as *P. malaccense* (Near‐Threatened), but also those currently considered safe, such as *C. erythropterum*, flagging them for regional conservation concern. Additionally, we find that even edge‐tolerant and seemingly well‐connected species, such as *M. gularis*, are likely impacted by fragmentation in the longer term (see also Tan et al., 2018). The great resolution provided by genomic analysis, even at extremely small spatial scales, can help understand population dynamics within protected areas, identify immediate threats and inform conservation at larger scales.

Barriers affecting understorey insectivorous birds undoubtedly impact other forest biota such as reptiles, amphibians and invertebrates that are known to be even more sensitive to habitat fragmentation (Keinath et al., 2017). Genomic studies such as ours can inform conservation efforts by identifying preferred locations for habitat corridors, flagging candidate populations for restocking with translocated individuals and improving the design of future reserves. Finally, this study provides a blueprint for conservation genomics at small scales, as the more commonly published regional scale studies may overlook critical local patterns.

## 5 CONCLUSION

What causes the continuing local extinction of species even in well‐protected areas such as Singapore's long‐established reserve network? Even in scenarios where total protected forest area remains stable, species loss will sometimes continue, as has been shown for Singapore (Yong, 2009). This study sheds light on this conundrum by demonstrating deep population genetic divisions in the wake of fragmentation over relatively short periods of time. By identifying fine‐scale geographic barriers to gene flow, genomic analyses can help restore population connectivity and prevent further local extinction. Once extirpated, a species may never recolonize in the absence of source populations, even when habitat becomes suitable again (Powell, Stouffer, & Johnson, 2013). It is therefore crucial to “protect locally while thinking globally.”

## Data Availability

The ddRADseq data have been deposited in GenBank: BioProject Accession Number: PRJNA598063.
