# Supplementary material for: Fine‐scale barriers to connectivity across a fragmented South‐East Asian landscape in six songbird species
Source: Evol Appl. 2020 Mar 5;13(5):1026–36. doi: 10.1111/eva.12918 (PMC7232758; doi:10.1111/eva.12918)
Supplement: Supplementary file 1 [file EVA-13-1026-s001.pdf]

Supporting Information

Table S1. Singapore samples of *Pellorneum malaccense*, *Cyanoderma erythropterum*, *Turdinus abbotti*, *Mixornis gularis*, *Pycnonotus plumosus*, and *Pycnonotus simplex* used in this study.

Species	Sample ID[a]	Date[b]	Sampling locality	Latitude	Longitude	Source[c]	GenBank sample name
P. malaccense	L2317	19/5/2015	North Central Catchment	1.381017	103.814667	this study	NUS-L2317
P. malaccense[d]	L2318	19/5/2015	North Central Catchment	1.381017	103.814667	this study	NUS-L2318
P. malaccense	L1530	8/10/2013	North Central Catchment	1.380551	103.810555	this study	NUS-L1530
P. malaccense	L1531	7/10/2013	North Central Catchment	1.380551	103.810555	this study	NUS-L1531
P. malaccense	L1569	4/6/2013	North Central Catchment	-	-	this study	NUS-L1569
P. malaccense	L1801	7/10/2013	North Central Catchment	1.380551	103.810555	this study	NUS-L1801
P. malaccense[d]	L1813	3/1/2014	North Central Catchment	1.383779	103.800888	this study	NUS-L1813
P. malaccense	L1814	3/1/2014	North Central Catchment	1.383779	103.800888	this study	NUS-L1814
P. malaccense	L1815	3/1/2014	North Central Catchment	1.383779	103.800888	this study	NUS-L1815
P. malaccense[d]	L1810	13/11/2013	North Central Catchment	1.377849	103.809926	this study	NUS-L1810
P. malaccense	L1809	5/11/2013	North Central Catchment	1.396403	103.803543	this study	NUS-L1809

P. malaccense	L1578	2/10/2013	North Central Catchment	1.396403	103.803543	this study	NUS-L1578
P. malaccense[d]	L1807	5/11/2013	North Central Catchment	1.396403	103.803543	this study	NUS-L1807
P. malaccense[d]	L2345	3/11/2015	North Central Catchment	1.398802	103.807574	this study	NUS-L2345
P. malaccense[e]	UWBM 81953	9/8/1998	North Central Catchment			BMNHC	NUS-UWBM 81953
P. malaccense[e]	UWBM 81952	9/8/1998	North Central Catchment			BMNHC	NUS-UWBM 81952
P. malaccense	L1565	20/4/2013	South Central Catchment	1.357036	103.812693	this study	NUS-L1565
P. malaccense	L1566	11/5/2013	South Central Catchment	1.357036	103.812693	this study	NUS-L1566
P. malaccense	UWBM 81955	13/8/1998	South Central Catchment			BMNHC	NUS-UWBM 81955
P. malaccense	UWBM 81957	14/8/1998	South Central Catchment			BMNHC	NUS-UWBM 81957
P. malaccense[e]	UWBM 81956	14/8/1998	South Central Catchment			BMNHC	NUS-UWBM 81956
P. malaccense	L1562	11/5/2013	South Central Catchment	1.357036	103.812693	this study	NUS-L1562
P. malaccense	L1838	26/3/2015	South Central Catchment	1.3552	103.813117	this study	NUS-L1838
P. malaccense	L1839	26/3/2015	South Central Catchment	1.3552	103.813117	this study	NUS-L1839
P. malaccense[d]	L1902	27/3/2015	South Central Catchment	1.359581	103.812837	this study	NUS-L1902
P. malaccense	L1808	5/11/2013	North Central Catchment	1.396403	103.803543	this study	NUS-L1808
P. malaccense	L1811	31/12/2013	South Central Catchment	1.351502	103.806386	this study	NUS-L1811

P. malaccense[d]	L1812	31/12/2013	South Central Catchment	1.351502	103.806386	this study	NUS-L1812
P. malaccense	L1816	7/1/2013	South Central Catchment	1.35595	103.801723	this study	NUS-L1816
P. malaccense	L1820	17/10/2013	South Central Catchment	1.355264	103.798923	this study	NUS-L1820
P. malaccense	L1821	8/1/2014	South Central Catchment	1.35595	103.801723	this study	NUS-L1821
P. malaccense	L1823	10/12/2014	South Central Catchment	1.35685	103.803733	this study	NUS-L1823
P. malaccense	L1824	10/12/2014	South Central Catchment	1.35615	103.803839	this study	NUS-L1824
P. malaccense	L1802	17/10/2013	South Central Catchment	1.355264	103.798923	this study	NUS-L1802
P. malaccense[d]	L1803	18/10/2013	South Central Catchment	1.355264	103.798923	this study	NUS-L1803
P. malaccense	L1827	3/3/2015	South Central Catchment	1.350917	103.829833	this study	NUS-L1827
P. malaccense	L1832	4/3/2015	South Central Catchment	1.35295	103.8265	this study	NUS-L1832
P. malaccense	L1833	5/3/2015	South Central Catchment	1.351767	103.83055	this study	NUS-L1833
P. malaccense	L1834	5/3/2015	South Central Catchment	1.351767	103.83055	this study	NUS-L1834
P. malaccense	L1836	5/3/2015	South Central Catchment	1.35225	103.828233	this study	NUS-L1836
P. malaccense	K0307	28/5/2015	South Central Catchment	1.352417	103.828617	this study	NUS-K0307
P. malaccense	L2321	28/5/2015	South Central Catchment	1.352417	103.828617	this study	NUS-L2321
P. malaccense[d]	L2325	29/5/2015	South Central Catchment	1.352583	103.827767	this study	NUS-L2325

P. malaccense	L1906	21/6/2014	South Central Catchment	1.348769	103.813112	this study	NUS-L1906
P. malaccense[d]	L1905	20/6/2014	South Central Catchment	1.348769	103.813112	this study	NUS-L1905
P. malaccense	M1132	19/8/2015	South Central Catchment	1.348607	103.803114	this study	NUS-M1132
P. malaccense[d]	L2326	18/8/2015	South Central Catchment	1.348607	103.803114	this study	NUS-L2326
P. malaccense	L1804	29/10/2013	South Central Catchment	1.353592	103.808224	this study	NUS-L1804
P. malaccense	L1806	30/10/2013	South Central Catchment	1.353592	103.808224	this study	NUS-L1806
P. malaccense[d]	L1805	30/10/2013	South Central Catchment	1.353592	103.808224	this study	NUS-L1805
P. malaccense	L2327	25/8/2015	South Central Catchment	1.35829	103.823675	this study	NUS-L2327
P. malaccense[d]	L1599	30/5/2014	South Central Catchment	1.356056	103.785652	this study	NUS-L1599
P. malaccense[d]	L1901	30/5/2014	South Central Catchment	1.356056	103.785652	this study	NUS-L1901
C. erythropterum	UWBM 117058	na	North Central Catchment	1.381017	103.814667	BMNHC	NUS-UWBM 117058
C. erythropterum	J3742	19/5/2015	North Central Catchment	1.381017	103.814667	this study	NUS-J3742
C. erythropterum	J3743	19/5/2015	North Central Catchment	1.381017	103.814667	this study	NUS-J3743
C. erythropterum[d]	J3744	19/5/2015	North Central Catchment	1.381017	103.814667	this study	NUS-J3744
C. erythropterum	K0642	22/5/2013	North Central Catchment	1.37744	103.807826	this study	NUS-K0642
C. erythropterum[d]	K0643	22/5/2013	North Central Catchment	1.37744	103.807826	this study	NUS-K0643

C. erythropterum	J63813	3/11/2015	North Central Catchment	1.397722	103.806375	this study	NUS-J63813
C. erythropterum[d]	J3817	24/11/2015	North Central Catchment	1.396329	103.802033	this study	NUS-J3817
C. erythropterum	J3736	26/3/2015	South Central Catchment	1.35924	103.812161	this study	NUS-J3736
C. erythropterum	J3737	26/3/2015	South Central Catchment	1.355	103.813017	this study	NUS-J3737
C. erythropterum	J3738	26/3/2015	South Central Catchment	1.355	103.813017	this study	NUS-J3738
C. erythropterum	K0625	11/5/2013	South Central Catchment	1.357036	103.812693	this study	NUS-K0625
C. erythropterum	J3734	26/3/2015	South Central Catchment	1.35924	103.812161	this study	NUS-J3734
C. erythropterum	J3735	26/3/2015	South Central Catchment	1.35924	103.812161	this study	NUS-J3735
C. erythropterum[d]	K0668	20/9/2013	South Central Catchment	1.357036	103.812693	this study	NUS-K0668
C. erythropterum	K1101	18/10/2013	South Central Catchment	1.355629	103.800891	this study	NUS-K1101
C. erythropterum	K1108	7/1/2014	South Central Catchment	1.355629	103.800891	this study	NUS-K1108
C. erythropterum	K1121	10/12/2014	South Central Catchment	1.355483	103.804517	this study	NUS-K1121
C. erythropterum	K1123	10/12/2014	South Central Catchment	1.35685	103.803733	this study	NUS-K1123
C. erythropterum[d]	K1124	11/12/2014	South Central Catchment	1.355367	103.8042	this study	NUS-K1124
C. erythropterum	K0690	21/6/2014	South Central Catchment	1.348769	103.813112	this study	NUS-K0690
C. erythropterum	D3315	3/3/2015	South Central Catchment	1.35295	103.8265	this study	NUS-D3315

C. erythropterum	J3725	3/3/2015	South Central Catchment	1.35295	103.8265	this study	NUS-J3725
C. erythropterum	J3724	3/3/2015	South Central Catchment	1.350917	103.829833	this study	NUS-J3724
C. erythropterum[d]	J3728	3/3/2015	South Central Catchment	1.350917	103.829833	this study	NUS-J3728
T. abbotti	L2308	7/4/2015	South Central Catchment	1.33815	103.817683	this study	NUS-L2308
T. abbotti	L2309	7/4/2015	South Central Catchment	1.33815	103.817683	this study	NUS-L2309
T. abbotti	L2314	9/4/2015	South Central Catchment	1.34085	103.820783	this study	NUS-L2314
T. abbotti	L2312	9/4/2015	South Central Catchment	1.34085	103.820783	this study	NUS-L2312
T. abbotti	L2315	9/4/2015	South Central Catchment	1.34085	103.820783	this study	NUS-L2315
T. abbotti	L2316	9/4/2015	South Central Catchment	1.34085	103.820783	this study	NUS-L2316
T. abbotti	L2313	9/4/2015	South Central Catchment	1.343267	103.821467	this study	NUS-L2313
T. abbotti	L1837	5/3/2015	South Central Catchment	1.35295	103.8265	this study	NUS-L1837
T. abbotti	L2322	28/5/2015	South Central Catchment	1.353433	103.8269	this study	NUS-L2322
T. abbotti	L2324	28/5/2015	South Central Catchment	1.353433	103.8269	this study	NUS-L2324
T. abbotti	L2323	28/5/2015	South Central Catchment	1.353433	103.8269	this study	NUS-L2323
T. abbotti	FL1826	17/12/2014	Sentosa Island	1.257096	103.813284	this study	NUS-FL1826
T. abbotti	L1738	21/8/2014	Ubin Island	1.412226	103.966195	this study	NUS-L1738

T. abbotti	L1718	22/8/2013	Ubin Island	1.412226	103.966195	this study	NUS-L1718
T. abbotti	L1719	22/8/2013	Ubin Island	1.412226	103.966195	this study	NUS-L1719
T. abbotti	M1466	20/8/2015	Ubin Island	1.419749	103.931458	this study	NUS-M1466
T. abbotti	M1460	25/6/2015	Ubin Island	1.419749	103.931458	this study	NUS-M1460
T. abbotti	FL1743	30/4/2015	Ubin Island	1.419749	103.931458	this study	NUS-FL1743
M. gularis	K1103	12/12/2013	Admiralty Park	1.449878	103.778439	Tan et al. 2018	NUS-K1103
M. gularis	K1104	12/12/2013	Admiralty Park	1.449878	103.778439	this study	NUS-K1104
M. gularis[d]	K1105	12/12/2013	Admiralty Park	1.449878	103.778439	this study	NUS-K1105
M. gularis	UWBM 117353	3/1/2007	North Central Catchment	1.345831	103.831843	BMNHC	NUS-UWBM 117353
M. gularis	J3137	7/3/2014	North Central Catchment	1.359081	103.782795	Tan et al. 2018	NUS-J3137
M. gularis[d]	K0679	7/3/2014	North Central Catchment	1.359081	103.782795	this study	NUS-K0679
M. gularis	J2843	7/3/2014	North Central Catchment	1.359081	103.782795	Tan et al. 2018	NUS-J2843
M. gularis	K0676	8/3/2014	North Central Catchment	1.359081	103.782795	Tan et al. 2018	NUS-K0676
M. gularis	K1111	10/1/2014	North Central Catchment	1.396495	103.803347	this study	NUS-K1111
M. gularis[d]	K0659	10/7/2013	North Central Catchment	1.398979	103.807907	this study	NUS-K0659
M. gularis	K0657	9/7/2013	North Central Catchment	1.398979	103.807907	this study	NUS-K0657

M. gularis	L1548	21/5/2013	North Central Catchment	1.37744	103.807826	Tan et al. 2018	NUS-L1548
M. gularis	K0640	21/5/2013	North Central Catchment	1.37744	103.807826	Tan et al. 2018	NUS-K0640
M. gularis	K0636	21/5/2013	North Central Catchment	1.37744	103.807826	Tan et al. 2018	NUS-K0636
M. gularis	K0670	4/12/2013	North Central Catchment	1.37744	103.807826	Tan et al. 2018	NUS-K0670
M. gularis	K0660	7/8/2013	North Central Catchment	1.372661	103.803922	Tan et al. 2018	NUS-K0660
M. gularis	K0646	4/6/2013	North Central Catchment	-	-	Tan et al. 2018	NUS-K0646
M. gularis	K0653	5/6/2013	North Central Catchment	-	-	Tan et al. 2018	NUS-K0653
M. gularis	K0655	5/6/2013	North Central Catchment	-	-	Tan et al. 2018	NUS-K0655
M. gularis	K1109	7/1/2014	South Central Catchment	1.35595	103.801723	Tan et al. 2018	NUS-K1109
M. gularis	K1122	10/12/2014	South Central Catchment	1.35685	103.803733	this study	NUS-K1122
M. gularis	K1107	31/12/2013	South Central Catchment	1.351502	103.806386	Tan et al. 2018	NUS-K1107
M. gularis	J3295	30/5/2014	North Central Catchment	1.356056	103.785652	Tan et al. 2018	NUS-J3295
M. gularis	J3502	31/5/2014	North Central Catchment	1.356056	103.785652	Tan et al. 2018	NUS-J3502
M. gularis	K0630	11/5/2013	South Central Catchment	1.357036	103.812693	Tan et al. 2018	NUS-K0630
M. gularis[d]	K0626	11/5/2013	South Central Catchment	1.357036	103.812693	this study	NUS-K0626
M. gularis	K0628	11/5/2013	South Central Catchment	1.357036	103.812693	Tan et al. 2018	NUS-K0628

M. gularis	K0662	24/8/2013	South Central Catchment	1.357036	103.812693	Tan et al. 2018	NUS-K0662
M. gularis	J38041	26/3/2015	South Central Catchment	1.3597	103.812817	this study	NUS-J38041
M. gularis[d]	K0684	25/4/2015	Bukit Timah Nature Reserve	1.35576	103.783022	this study	NUS-K0684
M. gularis	K0685	25/4/2015	Bukit Timah Nature Reserve	1.35576	103.783022	Tan et al. 2018	NUS-K0685
M. gularis	K0682	25/4/2015	Bukit Timah Nature Reserve	1.35576	103.783022	Tan et al. 2018	NUS-K0682
M. gularis	K0683	25/4/2015	Bukit Timah Nature Reserve	1.35576	103.783022	Tan et al. 2018	NUS-K0683
M. gularis	K0673	25/1/2014	Bukit Timah Nature Reserve	1.360476	103.777391	Tan et al. 2018	NUS-K0673
M. gularis	CR063	na	Kent Ridge Park	1.291595	103.781701	Tan et al. 2018	NUS-CR063
M. gularis	UWBM 117142	18/10/2006	Kent Ridge Park	1.286595	103.788739	BMNHC	NUS-UWBM 117142
M. gularis	UWBM 117174	18/10/2016	Kent Ridge Park	1.286595	103.788739	BMNHC	NUS-UWBM 117174
M. gularis	CSW8312	18/10/2006	Kent Ridge Park	1.286595	103.788739	Tan et al. 2018	NUS-CSW8312
M. gularis	CSW8313	18/10/2006	Kent Ridge Park	1.286595	103.788739	Tan et al. 2018	NUS-CSW8313
M. gularis	K1112	15/1/2014	Kent Ridge Park	1.286595	103.788739	Tan et al. 2018	NUS-K1112
M. gularis	K1113	15/1/2014	Kent Ridge Park	1.286595	103.788739	Tan et al. 2018	NUS-K1113
M. gularis[d]	K1114	30/1/2014	Kent Ridge Park	1.295293	103.777272	this study	NUS-K1114
M. gularis	K1106	18/12/2013	Clementi Woods Park	1.300877	103.766832	Tan et al. 2018	NUS-K1106

M. gularis	K1117	27/2/2014	Mt Faber Park	1.269568	103.821747	Tan et al. 2018	NUS-K1117
M. gularis	K1116	27/2/2014	Mt Faber Park	1.269568	103.821747	Tan et al. 2018	NUS-K1116
M. gularis	K1115	27/2/2014	Mt Faber Park	1.269568	103.821747	Tan et al. 2018	NUS-K1115
M. gularis[d]	K1102	7/11/2013	Telok Blangah Hill Park	1.278373	103.813504	this study	NUS-K1102
M. gularis	K1118	27/5/2014	Sentosa Island	1.257598	103.813652	Tan et al. 2018	NUS-K1118
M. gularis	K1119	27/5/2014	Sentosa Island	1.257598	103.813652	this study	NUS-K1119
M. gularis[d]	K1120	27/5/2014	Sentosa Island	1.257598	103.813652	this study	NUS-K1120
M. gularis	K1153	6/10/2015	Sentosa Island	1.35685	103.803733	this study	NUS-K1153
P. simplex[d]	L2320	20/5/2015	North Central Catchment	1.38135	103.814867	this study	NUS-L2320
P. simplex	L2319	20/5/2015	North Central Catchment	1.38135	103.814867	this study	NUS-L2319
P. simplex	K0652	4/6/2013	North Central Catchment	-	-	this study	NUS-K0652
P. simplex[d]	K0654	5/6/2013	North Central Catchment	-	-	this study	NUS-K0654
P. simplex[d]	K0664	3/9/2013	North Central Catchment	1.3817	103.815383	this study	NUS-K0664
P. simplex	K0665	4/9/2013	North Central Catchment	1.3817	103.815383	this study	NUS-K0665
P. simplex	K0666	4/9/2013	North Central Catchment	1.3817	103.815383	this study	NUS-K0666
P. simplex[d]	K0667	4/9/2013	North Central Catchment	1.3817	103.815383	this study	NUS-K0667

P. simplex[d]	L0674	30/5/2014	North Central Catchment	1.356056	103.785652	this study	NUS-L0674
P. simplex[d]	L1600	30/5/2014	North Central Catchment	1.356056	103.785652	this study	NUS-L1600
P. simplex[d]	K1155	3/11/2015	North Central Catchment	1.398462	103.806807	this study	NUS-K1155
P. simplex	K0658	10/7/2013	North Central Catchment	1.395656	103.804365	this study	NUS-K0658
P. simplex[d]	L2346	24/11/2015	North Central Catchment	1.397582	103.80457	this study	NUS-L2346
P. simplex[d]	L1825	11/12/2014	South Central Catchment	1.35685	103.803733	this study	NUS-L1825
P. simplex	L1828	3/3/2015	South Central Catchment	1.350917	103.829833	this study	NUS-L1828
P. simplex	L1829	3/3/2015	South Central Catchment	1.350917	103.829833	this study	NUS-L1829
P. simplex	L1831	4/3/2015	South Central Catchment	1.35295	103.8265	this study	NUS-L1831
P. simplex[d]	L1835	5/3/2015	South Central Catchment	1.35225	103.828233	this study	NUS-L1835
P. simplex[d]	M1125	3/3/2015	South Central Catchment	1.350917	103.829833	this study	NUS-M1125
P. simplex[d]	M1134	19/8/2015	South Central Catchment	1.348607	103.803114	this study	NUS-M1134
P. simplex	M1133	19/8/2015	South Central Catchment	1.348607	103.803114	this study	NUS-M1133
P. simplex[d]	L2301	25/3/2015	South Central Catchment	1.355	103.813017	this study	NUS-L2301
P. simplex[d]	K1133	17/11/2015	Bukit Timah Nature Reserve	1.350134	103.779752	this study	NUS-K1133
P. plumosus	M1109	13/12/2013	Admiralty Park	1.449878	103.778439	this study	NUS-M1109

P. plumosus[d]	M1111	13/12/2013	Admiralty Park	1.449878	103.778439	this study	NUS-M1111
P. plumosus	M0969	30/5/2014	North Central Catchment	1.356056	103.785652	this study	NUS-M0969
P. plumosus	M0968	30/5/2014	North Central Catchment	1.356056	103.785652	this study	NUS-M0968
P. plumosus	M0973	31/5/2014	North Central Catchment	1.356056	103.785652	this study	NUS-M0973
P. plumosus	M0972	31/5/2014	North Central Catchment	1.356056	103.785652	this study	NUS-M0972
P. plumosus	M0970	31/5/2014	North Central Catchment	1.356056	103.785652	this study	NUS-M0970
P. plumosus	L0655	30/5/2014	North Central Catchment	1.356056	103.785652	this study	NUS-L0655
P. plumosus	M0971	31/5/2014	North Central Catchment	1.356056	103.785652	this study	NUS-M0971
P. plumosus	M1113	3/1/2014	North Central Catchment	1.383779	103.800888	this study	NUS-M1113
P. plumosus	M0927	5/6/2013	North Central Catchment	-	-	this study	NUS-M0927
P. plumosus	M0935	4/9/2013	North Central Catchment	1.3817	103.815383	this study	NUS-M0935
P. plumosus	M0933	7/8/2013	North Central Catchment	1.372661	103.803922	this study	NUS-M0933
P. plumosus	M0932	7/8/2013	North Central Catchment	1.372661	103.803922	this study	NUS-M0932
P. plumosus	M0918	21/5/2013	North Central Catchment	1.37744	103.807826	this study	NUS-M0918
P. plumosus	M0922	22/5/2013	North Central Catchment	1.37744	103.807826	this study	NUS-M0922
P. plumosus	M0936	3/11/2015	North Central Catchment	1.397722	103.806375	this study	NUS-M0936

P. plumosus	M1114	9/1/2014	North Central Catchment	1.35595	103.801723	this study	NUS-M1114
P. plumosus	M0930	10/7/2013	North Central Catchment	1.398979	103.807907	this study	NUS-M0930
P. plumosus	L1589	6/9/2014	North Central Catchment	1.359081	103.782795	this study	NUS-L1589
P. plumosus[e]	K0651	4/6/2013	North Central Catchment	-	-	this study	NUS-K0651
P. plumosus	M0931	27/7/2013	South Central Catchment	1.357036	103.812693	this study	NUS-M0931
P. plumosus	L1568	11/5/2013	South Central Catchment	1.357036	103.812693	this study	NUS-L1568
P. plumosus	L1567	11/5/2013	South Central Catchment	1.357036	103.812693	this study	NUS-L1567
P. plumosus	M0999	20/6/2014	South Central Catchment	1.348769	103.813112	this study	NUS-M0999
P. plumosus	M1000	21/6/2014	South Central Catchment	1.348769	103.813112	this study	NUS-M1000
P. plumosus	M0031	21/6/2014	South Central Catchment	1.348769	103.813112	this study	NUS-M0031
P. plumosus	M1102	17/10/2013	South Central Catchment	1.355264	103.798923	this study	NUS-M1102
P. plumosus	L2310	7/4/2015	South Central Catchment	1.340867	103.820167	this study	NUS-L2310
P. plumosus	L2311	7/4/2015	South Central Catchment	1.3431	103.822317	this study	NUS-L2311
P. plumosus	L0975	25/3/2015	South Central Catchment	1.359549	103.812882	this study	NUS-L0975
P. plumosus[e]	M0914	21/9/2013	South Central Catchment	-	-	this study	NUS-M0914
P. plumosus	M1106	10/12/2013	Bukit Batok Nature Park	1.348233	103.762454	this study	NUS-M1106

P. plumosus[d]	M1105	10/12/2013	Bukit Batok Nature Park	1.348233	103.762454	this study	NUS-M1105
P. plumosus	M1107	10/12/2013	Bukit Batok Nature Park	1.348233	103.762454	this study	NUS-M1107
P. plumosus[e]	M1108	10/12/2013	Bukit Batok Nature Park	-	-	this study	NUS-M1108
P. plumosus	M0948	25/4/2014	Bukit Timah Nature Reserve	1.35576	103.783022	this study	NUS-M0948
P. plumosus	M0946	25/4/2014	Bukit Timah Nature Reserve	1.35576	103.783022	this study	NUS-M0946
P. plumosus	M0949	25/4/2014	Bukit Timah Nature Reserve	1.35576	103.783022	this study	NUS-M0949
P. plumosus	M0947	25/4/2014	Bukit Timah Nature Reserve	1.35576	103.783022	this study	NUS-M0947
P. plumosus	N0943	22/3/2014	Bukit Timah Nature Reserve	1.360476	103.777391	this study	NUS-N0943
P. plumosus	M0940	24/1/2014	Bukit Timah Nature Reserve	1.360476	103.777391	this study	NUS-M0940
P. plumosus	L0691	22/3/2014	Bukit Timah Nature Reserve	1.360476	103.777391	this study	NUS-L0691
P. plumosus	M1241	13/9/2014	Bukit Timah Nature Reserve	1.360476	103.777391	this study	NUS-M1241
P. plumosus	M0912	21/3/2014	Bukit Timah Nature Reserve	1.360476	103.777391	this study	NUS-M0912
P. plumosus	M0945	12/4/2014	Bukit Timah Nature Reserve	1.360476	103.777391	this study	NUS-M0945
P. plumosus	M0942	25/1/2014	Bukit Timah Nature Reserve	1.360476	103.777391	this study	NUS-M0942
P. plumosus	M1112	18/12/2013	Clementi Woods Park	1.300877	103.766832	this study	NUS-M1112
P. plumosus	M1117	24/2/2014	Mt Faber Park	1.269568	103.821747	this study	NUS-M1117

P. plumosus	M1116	24/2/2014	Mt Faber Park	1.269568	103.821747	this study	NUS-M1116
P. plumosus	M1103	31/10/2013	Mt Faber Park	1.269568	103.821747	this study	NUS-M1103
P. plumosus	M1104	31/10/2013	Mt Faber Park	1.269568	103.821747	this study	NUS-M1104
P. plumosus[d]	M1118	27/5/2014	Sentosa Island	1.257598	103.813652	this study	NUS-M1118
P. plumosus	M1144	6/10/2015	Sentosa Island	1.256867	103.813696	this study	NUS-M1144
P. plumosus[e]	UWBM 81977	29/12/1998	Sentosa Island	1.2603	103.8281	BMNHC	NUS-UWBM 81977
P. plumosus	UWBM 116734	4/5/2012	Semakau Island	1.20706	103.759081	BMNHC	NUS-UWBM 116734
P. plumosus	UWBM 116735	6/5/2012	Semakau Island	1.20706	103.759081	BMNHC	NUS-UWBM 116735
P. plumosus	UWBM 116736	6/5/2012	Semakau Island	1.20706	103.759081	BMNHC	NUS-UWBM 116736
P. plumosus	UWBM 116737	6/5/2012	Semakau Island	1.20706	103.759081	BMNHC	NUS-UWBM 116737
P. plumosus	UWBM 116738	5/5/2012	Semakau Island	1.20706	103.759081	BMNHC	NUS-UWBM 116738
P. plumosus	UWBM 116739	5/5/2012	Semakau Island	1.20706	103.759081	BMNHC	NUS-UWBM 116739
P. plumosus	UWBM 116730	4/5/2012	Semakau Island	1.20706	103.759081	BMNHC	NUS-UWBM 116730
P. plumosus	UWBM 117299	5/5/2012	Semakau Island	1.20706	103.759081	BMNHC	NUS-UWBM 117299
P. plumosus	UWBM 81971	4/9/1998	Semakau Island	1.20706	103.759081	BMNHC	NUS-UWBM 81971
P. plumosus	UWBM 83479	8/4/2005	Semakau Island	1.20706	103.759081	BMNHC	NUS-UWBM 83479

P. plumosus	UWBM 89181	12/5/2004	Semakau Island	1.20706	103.759081	BMNHC	NUS-UWBM 89181
P. plumosus	UWBM116727	na	Semakau Island	1.20706	103.759081	BMNHC	NUS-UWBM116727
P. plumosus	UWBM 116732	na	Semakau Island	1.20706	103.759081	BMNHC	NUS-UWBM 116732
P. plumosus	UWBM 116733	na	Semakau Island	1.20706	103.759081	BMNHC	NUS-UWBM 116733
P. plumosus[d]	M0834	22/8/2013	Ubin Island	1.412226	103.966195	this study	NUS-M0834
P. plumosus	M1402	21/8/2014	Ubin Island	1.412226	103.966195	this study	NUS-M1402
P. plumosus	M0832	22/8/2013	Ubin Island	1.412226	103.966195	this study	NUS-M0832
P. plumosus	M0830	22/8/2013	Ubin Island	1.412226	103.966195	this study	NUS-M0830
P. plumosus	N0836	22/8/2013	Ubin Island	1.412226	103.966195	this study	NUS-N0836
P. plumosus	L1736	26/6/2014	Ubin Island	1.412226	103.966195	this study	NUS-L1736
P. plumosus[e]	M0831	22/8/2013	Ubin Island	-	-	this study	NUS-M0831
P. plumosus[e]	UWBM 82005	10/5/1999	Tekong Island	1.4051	104.0503	BMNHC	NUS-UWBM 82005
P. plumosus[e]	UWBM 67504	22/5/2001	Sungei Buloh Nature Park	1.4448	103.7258	BMNHC	NUS-UWBM 67504

[a]Refers to voucher numbers for samples from museum collections and to field numbers for samples specifically collected for this study

[b]Refers to sampling date in the field

[c]Indicates “this study” for samples mist-netted, blood-sampled, ringed and released specifically for this project; otherwise museum source is provided: BMNHC – Burke Museum of Natural History and Culture, Seattle, Washington; LKCNHM – Lee Kong Chian Natural History Museum, Singapore. ”Tan et al. 2018” for samples sequenced a part of this published study.

[d]Denotes samples additionally used in the comparative analysis with contiguous forest

[e]Denotes samples discarded after quality check

Table S2. Non-Singaporean samples of *Pellorneum malaccense*, *Cyanoderma erythropterum*, *Mixornis gularis*, *Pycnonotus plumosus*, and *Pycnonotus simplex* used in this study.

Species	Sample ID[a]	Country	Sampling locality	Source[b]	GenBank sample name
P. malaccense	B2002	Malaysia	Sabah, Danum Valley	this study	NUS-B2002
P. malaccense	B2042	Malaysia	Sabah, Danum Valley	this study	NUS-B2042
P. malaccense	B2046	Malaysia	Sabah, Danum Valley	this study	NUS-B2046
P. malaccense	B2053	Malaysia	Sabah, Danum Valley	this study	NUS-B2053
P. malaccense	B2057	Malaysia	Sabah, Danum Valley	this study	NUS-B2057
P. malaccense	B2113	Malaysia	Sabah, Danum Valley	this study	NUS-B2113
P. malaccense	B2114	Malaysia	Sabah, Danum Valley	this study	NUS-B2114
P. malaccense	B2203	Malaysia	Sabah, Danum Valley	this study	NUS-B2203
P. malaccense	B2206	Malaysia	Sabah, Danum Valley	this study	NUS-B2206
P. malaccense	B2228	Malaysia	Sabah, Danum Valley	this study	NUS-B2228
P. malaccense	B2243	Malaysia	Sabah, Danum Valley	this study	NUS-B2243
P. malaccense	B2245	Malaysia	Sabah, Danum Valley	this study	NUS-B2245
P. malaccense	B2551	Malaysia	Sabah, Danum Valley	this study	NUS-B2551

P. malaccense	B2594	Malaysia	Sabah, Danum Valley	this study	NUS-B2594
C. erythropterum	A2939	Malaysia	Sabah, Danum Valley	this study	NUS-A2939
C. erythropterum	A2942	Malaysia	Sabah, Danum Valley	this study	NUS-A2942
C. erythropterum	A3061	Malaysia	Sabah, Danum Valley	this study	NUS-A3061
C. erythropterum	A3405	Malaysia	Sabah, Danum Valley	this study	NUS-A3405
C. erythropterum	A3422	Malaysia	Sabah, Danum Valley	this study	NUS-A3422
C. erythropterum	B2121	Malaysia	Sabah, Danum Valley	this study	NUS-B2121
M. gularis	NRM20046847	Vietnam	Quang Tri, Dakrong Bridge	SMNH	NUS-NRM20046847
M. gularis	NRM 20046881	Vietnam	Quang Tri, Dakrong Bridge	SMNH	NUS-NRM 20046881
M. gularis	NRM 20046895	Vietnam	Quang Tri, Dakrong Bridge	SMNH	NUS-NRM 20046895
M. gularis	NRM 20046910	Vietnam	Quang Tri, Dakrong Bridge	SMNH	NUS-NRM 20046910
M. gularis	NRM 20046920	Vietnam	Quang Tri, Dakrong Bridge	SMNH	NUS-NRM 20046920
M. gularis	NRM 20046948	Vietnam	Quang Tri, Dakrong Bridge	SMNH	NUS-NRM 20046948
M. gularis	NRM 20046968	Vietnam	Quang Tri, Dakrong, A. Dang	SMNH	NUS-NRM 20046968
M. gularis	NRM 20047044	Vietnam	Quang Tri, Dakrong, A. Dang	SMNH	NUS-NRM 20047044
P. simplex	UWBM 81914	Malaysia	Sarawak, Bako National Park	BMNHC	NUS-UWBM 81914

P. simplex	UWBM 81919	Malaysia	Sarawak, Bako National Park	BMNHC	NUS-UWBM 81919
P. simplex	UWBM 81923	Malaysia	Sarawak, Bako National Park	BMNHC	NUS-UWBM 81923
P. simplex	UWBM 81924	Malaysia	Sarawak, Bako National Park	BMNHC	NUS-UWBM 81924
P. simplex	UWBM 81925	Malaysia	Sarawak, Bako National Park	BMNHC	NUS-UWBM 81925
P. simplex	UWBM 81927	Malaysia	Sarawak, Bako National Park	BMNHC	NUS-UWBM 81927
P. simplex	UWBM 81928	Malaysia	Sarawak, Bako National Park	BMNHC	NUS-UWBM 81928
P. simplex	UWBM 82062	Malaysia	Sarawak, Bako National Park	BMNHC	NUS-UWBM 82062
P. simplex	UWBM 82072	Malaysia	Sarawak, Bako National Park	BMNHC	NUS-UWBM 82072
P. simplex	UWBM 82073	Malaysia	Sarawak, Bako National Park	BMNHC	NUS-UWBM 82073
P. simplex	UWBM 82085	Malaysia	Sarawak, Bako National Park	BMNHC	NUS-UWBM 82085
P. simplex	UWBM 82088	Malaysia	Sarawak, Bako National Park	BMNHC	NUS-UWBM 82088
P. simplex	UWBM 82089	Malaysia	Sarawak, Bako National Park	BMNHC	NUS-UWBM 82089
P. simplex	UWBM 82090	Malaysia	Sarawak, Bako National Park	BMNHC	NUS-UWBM 82090
P. plumosus	UWBM 81909	Malaysia	Sarawak, Bako National Park	BMNHC	NUS-UWBM 81909
P. plumosus	UWBM 81915	Malaysia	Sarawak, Bako National Park	BMNHC	NUS-UWBM 81915
P. plumosus	UWBM 81934	Malaysia	Sarawak, Bako National Park	BMNHC	NUS-UWBM 81934

P. plumosus	UWBM 81936	Malaysia	Sarawak, Bako National Park	BMNHC	NUS-UWBM 81936
P. plumosus[c]	UWBM 81913	Malaysia	Sarawak	BMNHC	NUS-UWBM 81913

[a]Refers to voucher numbers for samples from museum collections and to field numbers for samples specifically collected for this study

[b]Indicates “this study” for samples mist-netted, blood-sampled, ringed and released specifically for this project; otherwise museum source is provided: BMNHC – Burke Museum of Natural History and Culture, Seattle, Washington; LKCNHM – Lee Kong Chian Natural History Museum, Singapore

[c]Denotes samples discarded after quality check

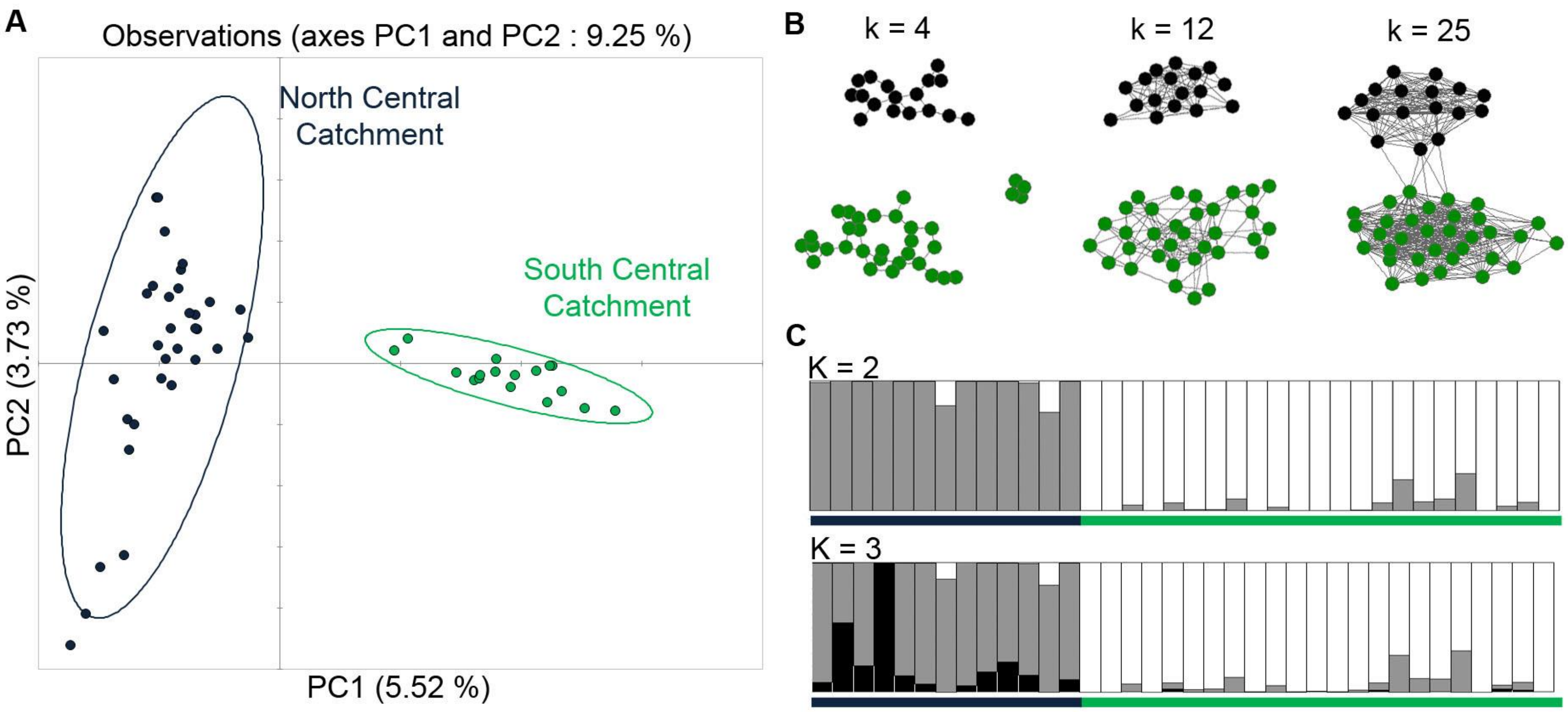

Fig. S1. Population structure of *Pellorneum* malaccense in Singapore. A: Principal component (PC) analysis of SNP data with a 95% confidence ellipse; B: Population clustering based on network analysis at k=4, 12, 25. Dot color indicates sampling localities; C: STRUCTURE analysis at K=2 (optimal K from Evanno's method, see Methods section 2.5) and at K=3. Horizontal bar color indicates sampling localities (see Supporting Information for sample ID and information).

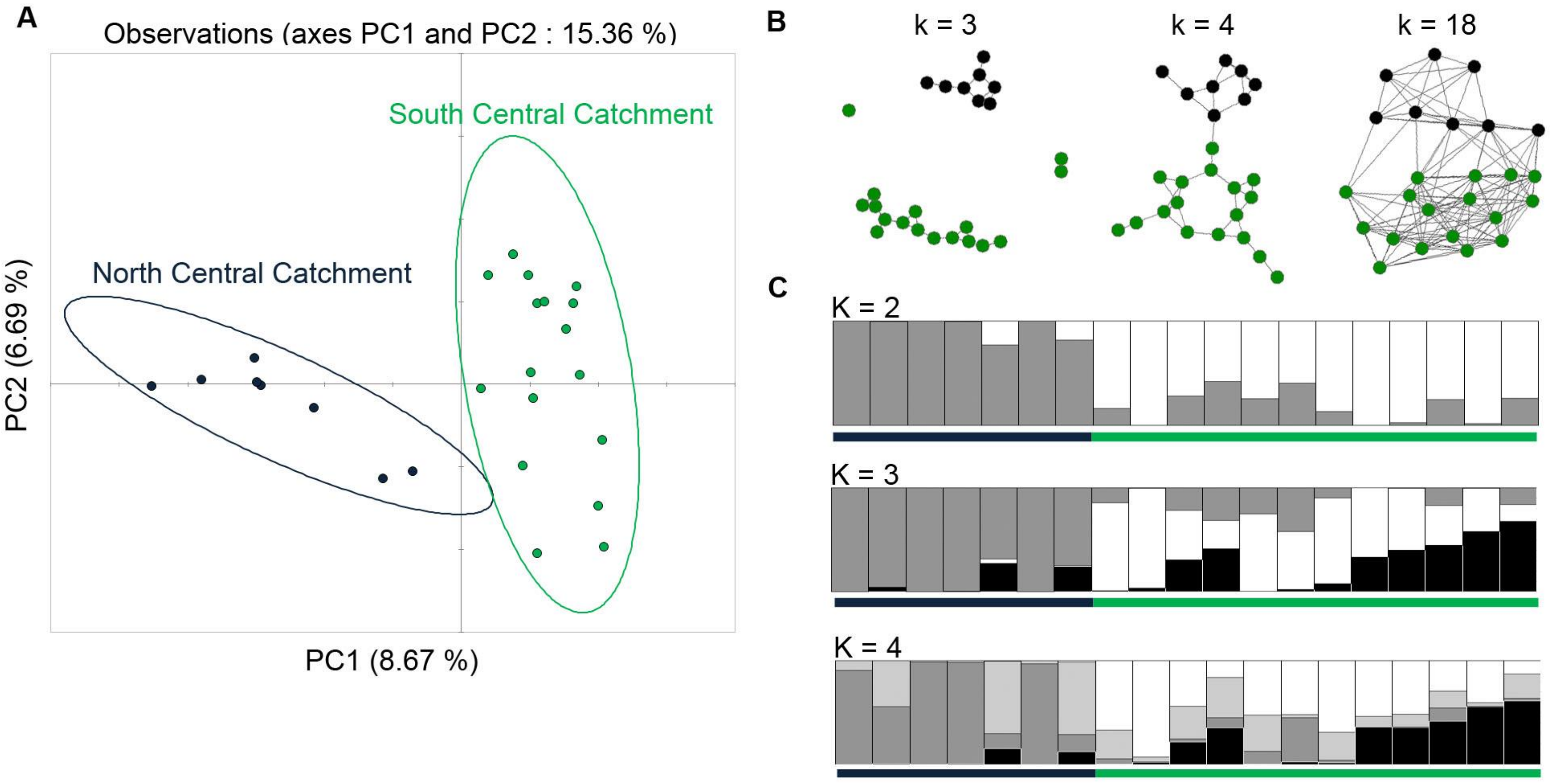

Fig. S2. Population structure of *Cyanoderma erythropterum* in Singapore. A: Principal component (PC) analysis of SNP data with a 95% confidence ellipse; B: Population clustering based on network analysis at k=3, 4, 18. Dot color indicates sampling localities; C: STRUCTURE analysis at K=2-3 and at K=4 (optimal K from Evanno's method, see Methods section 2.5). Horizontal bar color indicates sampling localities (see Supporting Information for sample ID and information).

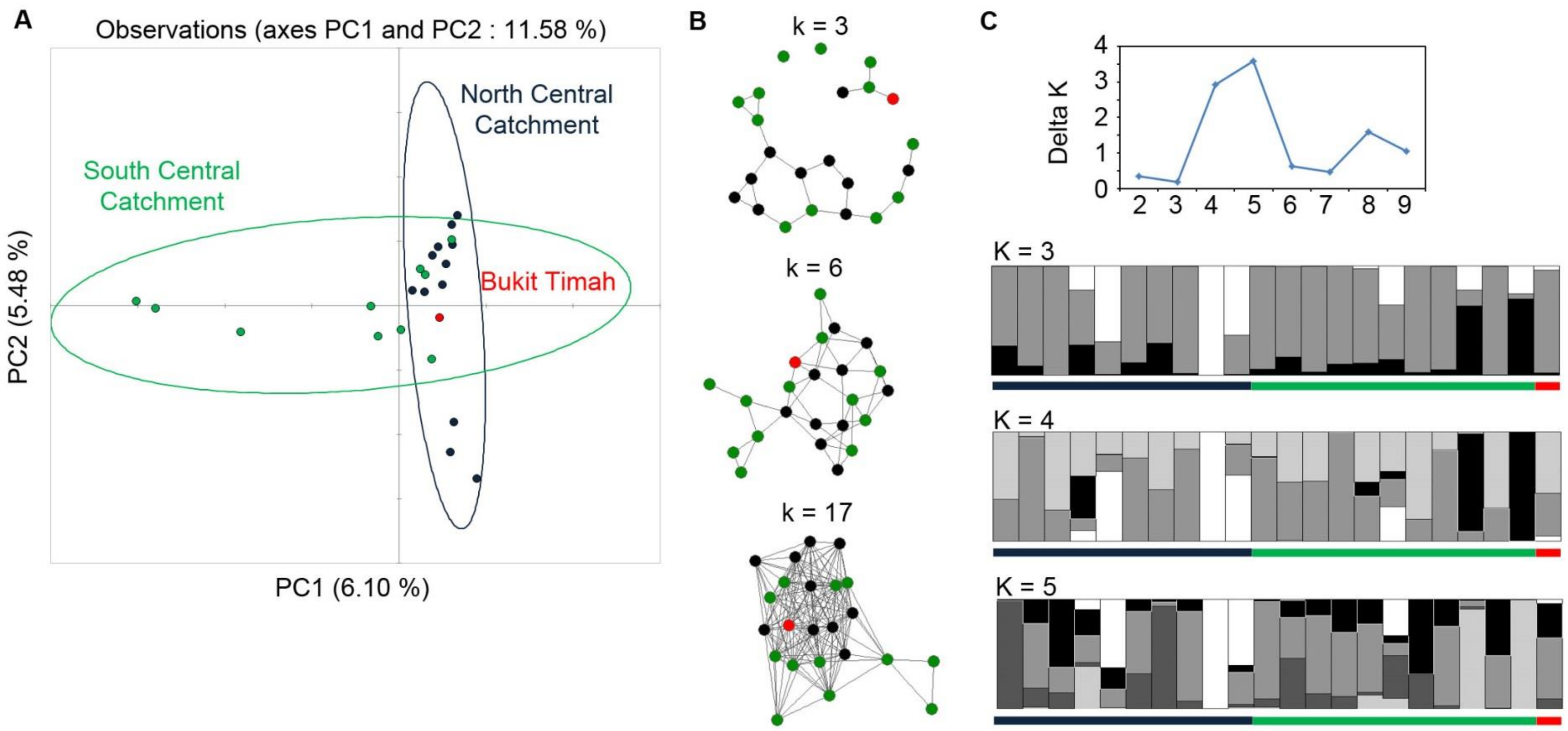

Fig. S3. Population structure of *Pycnonotus simplex* in Singapore. A: Principal component (PC) analysis of SNP data with a 95% confidence ellipse; B: Population clustering based on network analysis at k=3, 6, 17. Dot color indicates sampling localities; C: STRUCTURE analysis at K=3-5. Evanno's Delta K (see Methods section 2.5) values are low and suggest no considerable population structure. Horizontal bar color indicates sampling localities (see Supporting Information for sample ID and information).

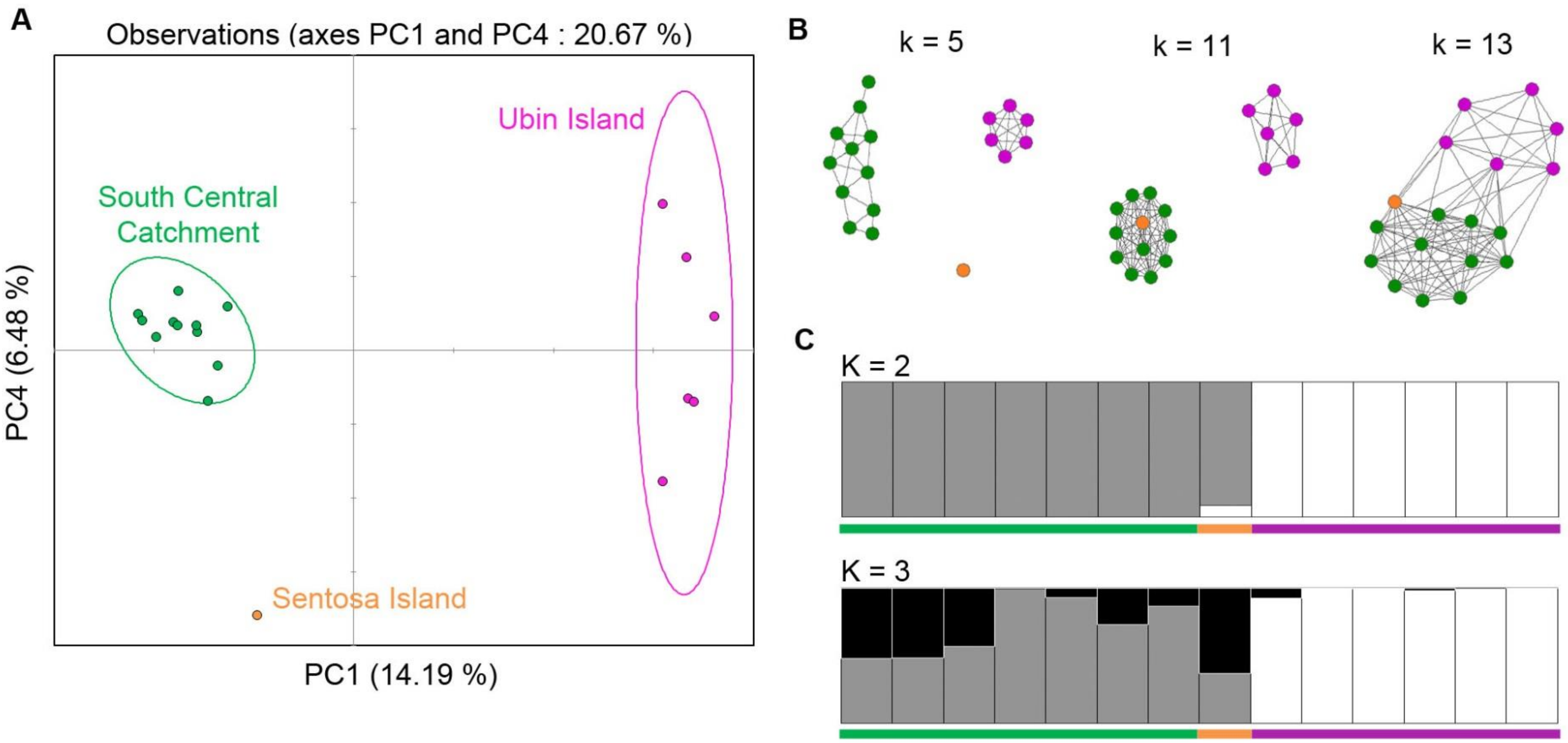

Fig. S4. Population structure of *Turdinus abbotti* in Singapore. A: Principal component (PC) analysis of SNP data with a 95% confidence ellipse; B: Population clustering based on network analysis at k=5, 11, 13. Dot color indicates sampling localities; C: STRUCTURE analysis at K=2 (optimal K from Evanno's method, see Methods section 2.5) and K=3. Horizontal bar color indicates sampling localities (see Supporting Information for sample ID and information).

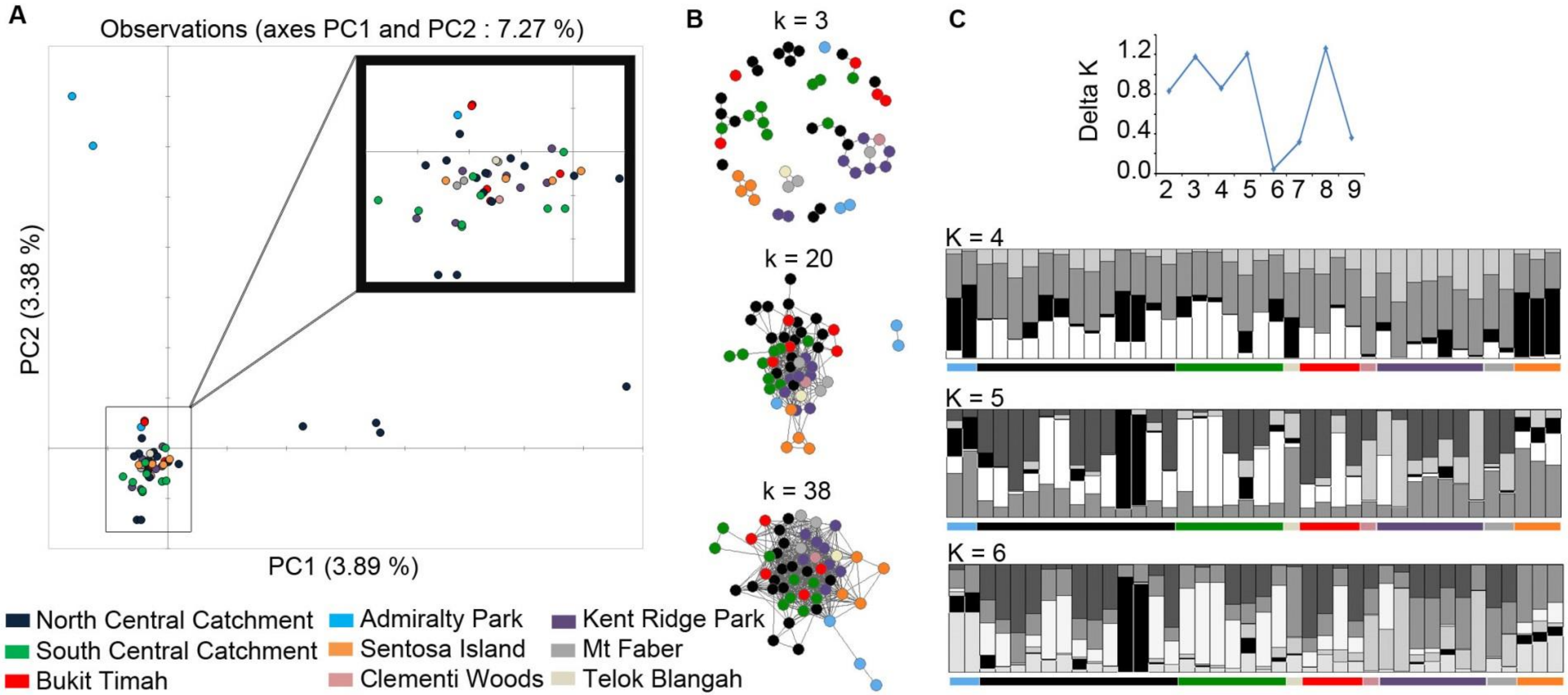

Fig. S5. Population structure of *Mixornis gularis* in Singapore. A: Principal component (PC) analysis of SNP data with a 95% confidence ellipse; B: Population clustering based on network analysis at k=3, 20, 38. Dot color indicates sampling localities; C: STRUCTURE analysis at K=4-6. Evanno's Delta K values (see Methods section 2.5) are low and suggest no considerable population structure. Horizontal bar color indicates sampling localities (see Supporting Information for sample ID and information).

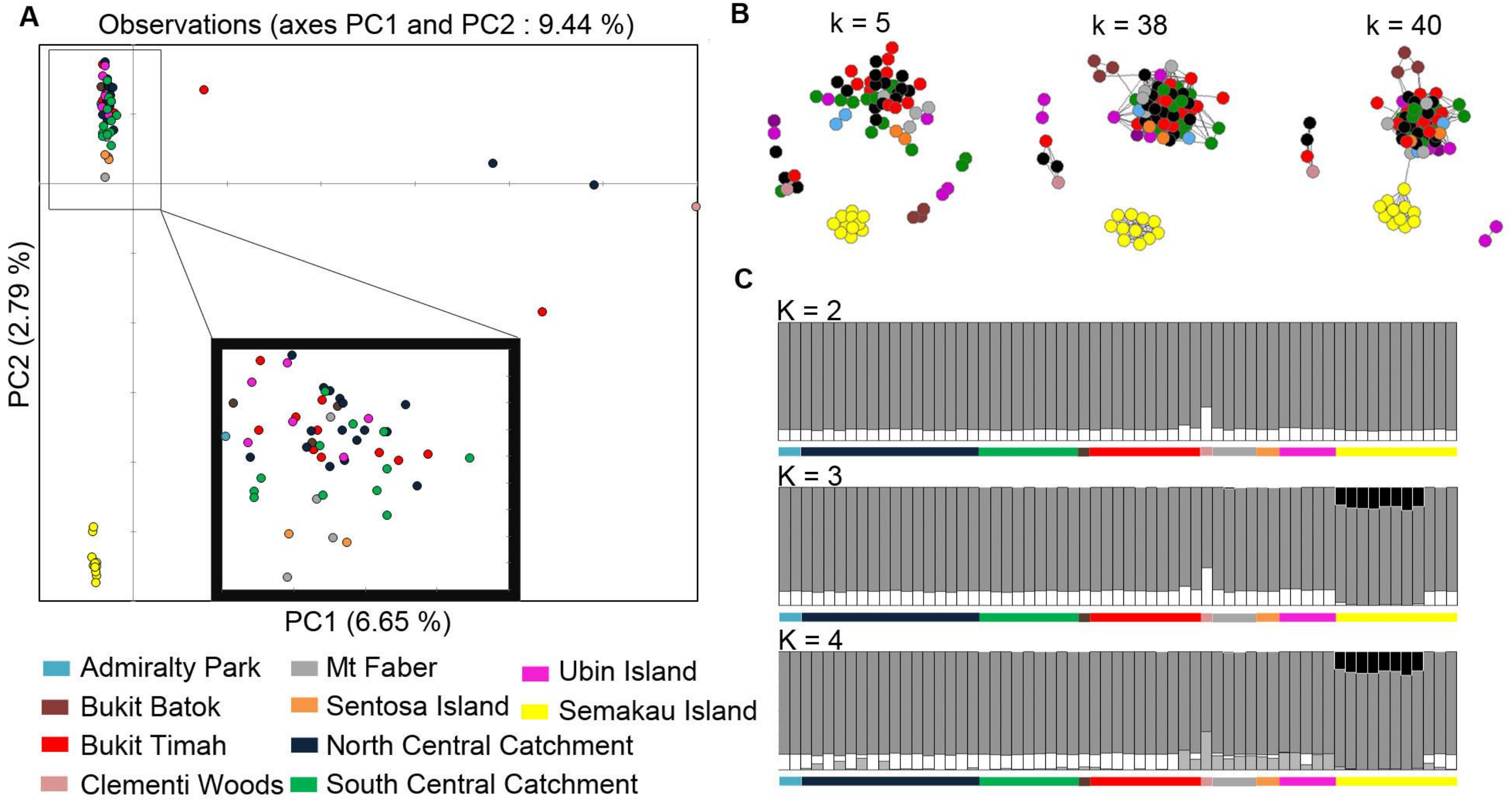

Fig. S6. Population structure of *Pycnonotus plumosus* in Singapore. A: Principal component (PC) analysis of SNP data with a 95% confidence ellipse; B: Population clustering based on network analysis at k=5, 38, 40. Dot color indicates sampling localities; C: STRUCTURE analysis at K=2-3 and at K=4 (optimal K from Evanno's method, see Methods section 2.5). Horizontal bar color indicates sampling localities (see Supporting Information for sample ID and information).

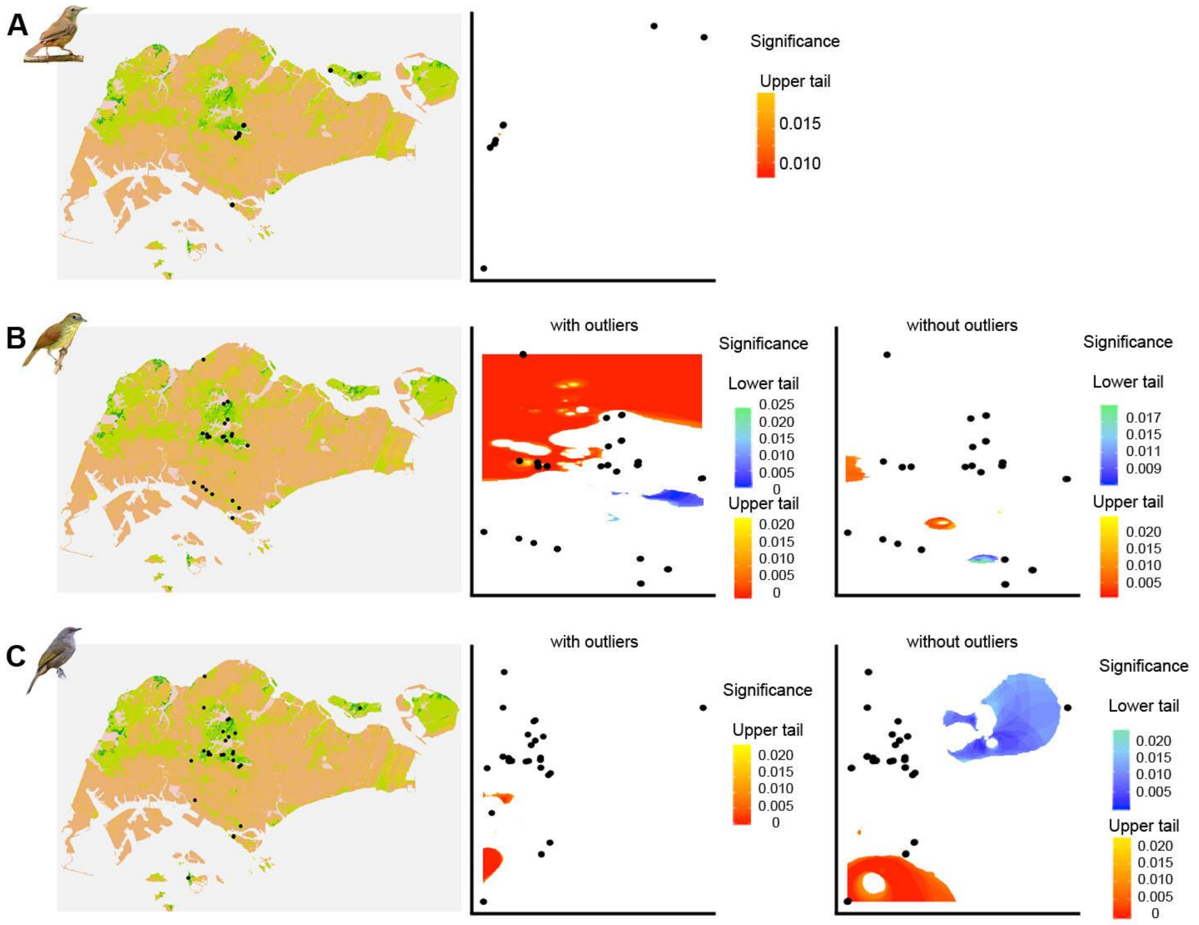

Fig. S7. Barriers to and conduits for gene flow in (A) *Turdinus abbotti*, (B) *Mixornis gularis* and (C) *Pycnonotus plumosus* using spatially explicit individual based analysis. Significant deviations ($p<0.05$) from assumptions under isolation by distance are shown in hues of red and indicate the presence of areas between individuals separated by a comparatively high genetic distance, constituting potential barriers. A genetic similarity significantly higher than expected from isolation by distance is shown in hues of blue, indicating areas between individuals separated by comparatively low genetic distance, constituting potential corridors for gene flow (see Supporting Information for sample ID and information). Bird pictures courtesy of Daniel Koh.

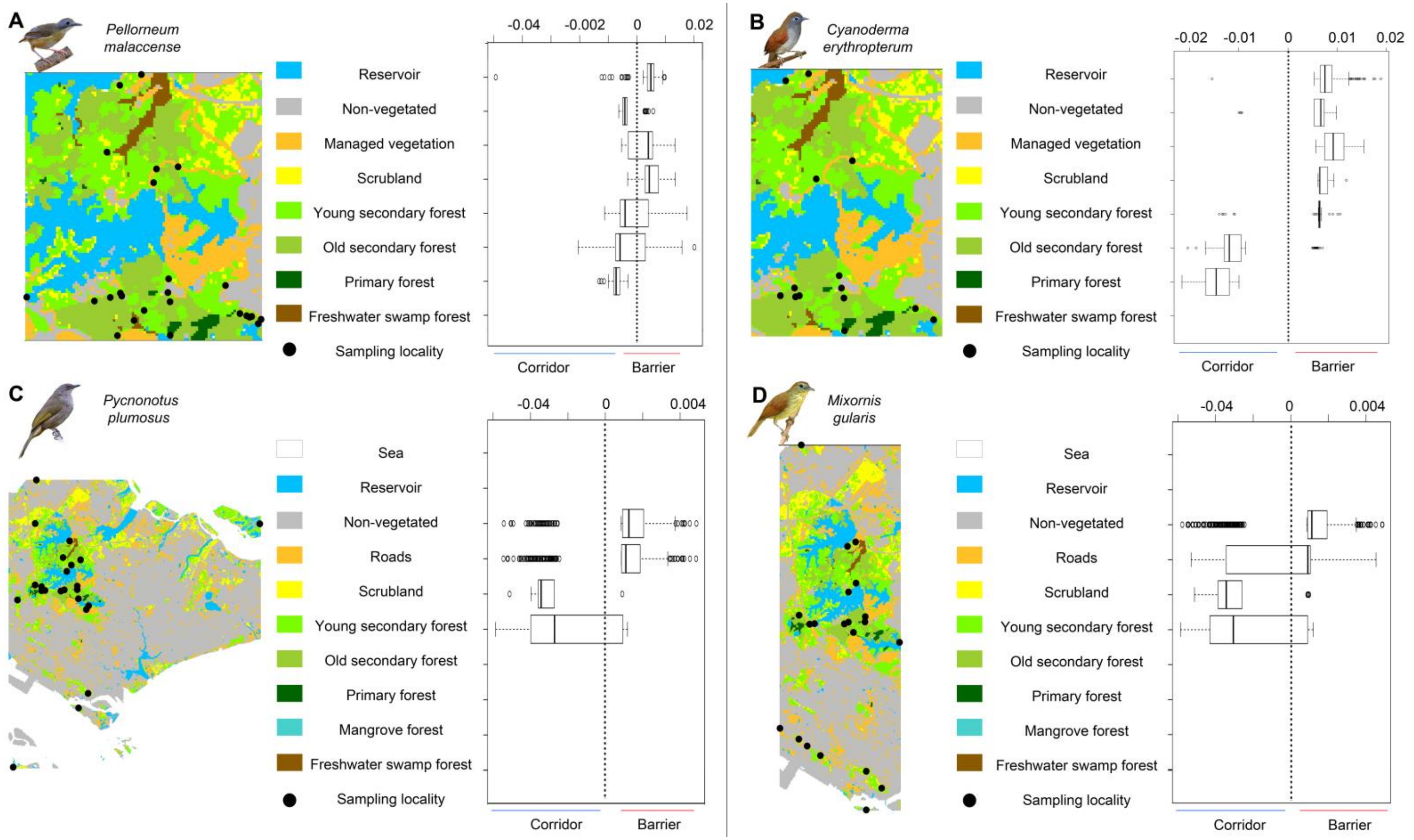

Fig. S8. Level of resistance of landscape features for *(A) Pellorneum malaccense, (B) Cyanoderma erythropterum, (C) Pycnonotus plumosus, and (D) Mixornis gularis* in Singapore. The maps (modified from Yee, Corlett, Liew, & Tan (2011) show landscape features and sampling localities. The box plots indicate the level of resistance of each landscape feature, with positive values indicating potential barriers and negative values potential corridors. Bird pictures courtesy of Daniel Koh.

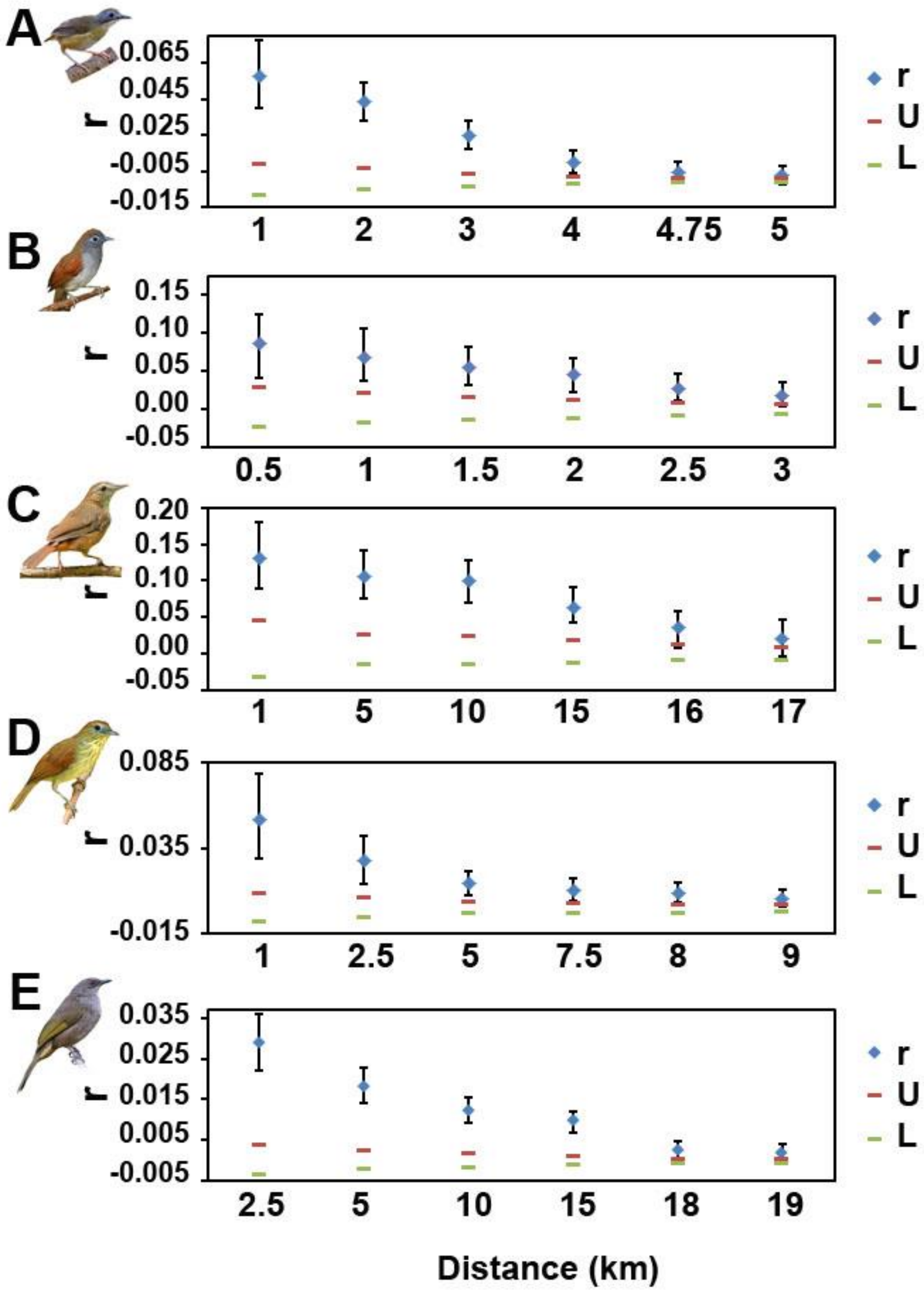

Fig. S9. Spatial autocorrelation for (A) *Pellorneum malaccense*, (B) *Cyanoderma erythropterum*, (C) *Turdinus abbotti*, (D) *Mixornis gularis* and (E) *Pycnonotus plumosus*. The graph shows the correlation r at different distance class sizes with 95% confidence error bars determined by bootstrap resampling, the upper (U), and lower (L) bounds for the 95% confidence interval of the null hypothesis of no spatial structure r=0. Bird pictures courtesy of Daniel Koh.
